# Identification of Potential Biomarkers in Association With Progression and Prognosis in Epithelial Ovarian Cancer by Integrated Bioinformatics Analysis

**DOI:** 10.3389/fgene.2019.01031

**Published:** 2019-10-24

**Authors:** Jinhui Liu, Huangyang Meng, Siyue Li, Yujie Shen, Hui Wang, Wu Shan, Jiangnan Qiu, Jie Zhang, Wenjun Cheng

**Affiliations:** ^1^Department of Gynecology, The First Affiliated Hospital of Nanjing Medical University, Nanjing, China; ^2^Department of Otorhinolaryngology, The First Affiliated Hospital of Nanjing Medical University, Nanjing, China

**Keywords:** epithelial ovarian cancer, bioinformatical analysis, differentially expressed genes, prognosis, Cmap, protein–protein interaction, biomarker

## Abstract

Epithelial ovarian cancer (EOC) is one of the malignancies in women, which has the highest mortality. However, the microlevel mechanism has not been discussed in detail. The expression profiles GSE27651, GSE38666, GSE40595, and GSE66957 including 188 tumor and 52 nontumor samples were downloaded from the Gene Expression Omnibus database. The differentially expressed genes (DEGs) were filtered using R software, and we performed functional analysis using the clusterProfiler. Cytoscape software, the molecular complex detection plugin and database STRING analyzed DEGs to construct protein-protein interaction network. We identified 116 DEGs including 81 upregulated and 35 downregulated DEGs. Functional analysis revealed that they were significantly enriched in the extracellular region and biosynthesis of amino acids. We next identified four bioactive compounds (vorinostat, LY-294002,trichostatin A, and tanespimycin) based on ConnectivityMap. Then 114 nodes were obtained in protein–protein interaction. The three most relevant modules were detected. In addition, according to degree ≥ 10, 14 core genes including FOXM1, CXCR4, KPNA2, NANOG, UBE2C, KIF11, ZWINT, CDCA5, DLGAP5, KIF15, MCM2, MELK, SPP1, and TRIP13 were identified. Kaplan–Meier analysis, Oncomine, and Gene Expression Profiling Interactive Analysis showed that overexpression of FOXM1, SPP1, UBE2C, KIF11, ZWINT, CDCA5, UBE2C, and KIF15 was related to bad prognosis of EOC patients. CDCA5, FOXM1, KIF15, MCM2, and ZWINT were associated with stage. Receiver operating characteristic (ROC) curve showed that messenger RNA levels of these five genes exhibited better diagnostic efficiency for normal and tumor tissues. The Human Protein Atlas database was performed. The protein levels of these five genes were significantly higher in tumor tissues compared with normal tissues. Functional enrichment analysis suggested that all the hub genes played crucial roles in citrate cycle tricarboxylic acid cycle. Furthermore, the univariate and multivariate Cox proportional hazards regression showed that ZWINT was independent prognostic indictor among EOC patients. The genes and pathways discovered in the above studies may open a new direction for EOC treatment.

## Introduction

Ovarian cancer is the second most common female malignant tumor in the world and the most common cause of death among female malignant tumors (McAlpine et al., 2014). With the development of the times, although surgery and other treatment methods have been improved, the treatment effect and prognosis of advanced ovarian cancer patients are very poor due to the difficulty in the diagnosis of ovarian cancer (Allemani et al., 2015; La Vecchia, 2017).

Gene expression microarray, as a means of efficient large-scale acquisition of genetic data, has been generally used to collect and study gene chip expression profiling data of many human cancers. New methods are provided by microarrays for studying tumor-associated genes, molecular targeting, molecular prediction, and therapy. The integration of databases where researchers have published their research data containing several gene expression chips allows for a more in-depth study of molecular mechanisms (Nannini et al., 2009; Petryszak et al., 2014).

In this study, we downloaded four gene expression datasets, GSE27651, GSE38666, GSE40595, and GSE66957 from The National Center for Biotechnology Information Gene Expression Omnibus (GEO) database. R software and Bioconductor software package was used to integrate chip data, combined with R package clusterProfiler, to mine gene ontology (GO) and Kyoto Encyclopedia of Genes and Genomes (KEGG) enrichment pathway. The core genes were screened from the protein–protein interaction (PPI) network of differentially expressed genes (DEGs). Finally, survival analysis was performed using a Kaplan–Meier plotter to further validate core genes. The genes and pathways discovered in the above studies may open a new direction for EOC treatment.

## Material and Methods

### Data Collection and Data Preprocessing

The raw data for GSE27651, GSE38666, GSE40595, and GSE66957 were integrated for the analysis. The gene chip was obtained from the GEO database (http://www.ncbi.nlm.nih.gov/geo/). GSE27651 included 43 cancer tissues and 6 normal tissues, dataset GSE38666 included 25 cancer tissues and 20 normal tissues, dataset GSE40595 included 63 cancer tissues and 14 normal tissues, and dataset GSE66957 included 57 cancer tissues and 12 normal tissues. They were functioned by Affymetrix Human Genome U133 Plus 2.0 Array [transcript (gene) version] (Affymetrix, Santa Clara, CA, USA) (Harbig et al., 2005). Robust multiarray average approach was performed for background correction and normalization (Irizarry et al., 2003). The original GEO data was then converted into expression measures using affy R package (Gautier et al., 2004).

### Differentially Expressed Genes

The “limma” R language package was utilized to detect the DEGs between EOC samples and normal samples in GEO database (Ritchie et al., 2015). We set the adjusted *P* < 0.05 and |log2 fold change (FC)| )1 as the cutoff criteria. Online Wayne diagram was used for identifying the common DEGs among GSE27651, GSE38666, GSE40595, and GSE66957. The drawing of the heatmap was done through the “heatmap” package of R 3.4.4.(Galili et al., 2018)

### GO Term and KEGG Pathway Enrichment Analysis

The function and pathway enrichment of the candidate genes were analyzed and annotated using the DAVID database (https://david.ncifcrf.gov/). GO annotations were performed on the screened DEGs using the DAVID online tool and clusterProfiler (Yu et al., 2012). Analysis of KEGG pathway of DEGs was performed using clusterProfiler. We set *p* < 0.05 as a significant criterion.

### Comprehensive Analysis of PPI Network and Functional Analysis

STRING (http://www.string-db.org/) was used to assess PPI information (Szklarczyk et al., 2015). In addition, Cytoscape software visualized the results to show the relationship between DEGs. The molecular complex detection (MCODE) plugin was used to search for cluster subnets. We used the following parameters: node score cutoff = 0.2, degree cutoff = 2, max. depth = 100 and k-core = 2. We further used the clusterProfiler to perform functional analysis of the genes in the hub module.

### Identification of Potential Drugs

The EOC gene signature was used to query ConnectivityMap (CMap) to find potential drugs for use in patients. The CMap database is a computer simulation method of predicting the potential drugs that may induce or reverse a biological state that encoded by the gene expression signature (Lamb et al., 2006). The different probe components commonly found between EOC tissue samples and normal tissue samples, then used to search the CMap database, are divided into the up- and downregulated groups. An enrichment score representing similarity is finally calculated. The positive connectivity score illustrates that the drug is capable of inducing cancer in human. On the contrary, the negative link score illustrates that the drug is able to reverse the cancer procedure. The negative connectivity score was indicated potential therapeutic value. Tomographs of these candidate molecular drugs were investigated in Pubchem database https://pubchem.ncbi.nlm.nih.gov/).

### Validation of Hub Genes

To find key genes that play an important role in EOC, we used Gene Expression Profiling Interactive Analysis (GEPIA) and Kaplan–Meier analysis to analyze the expression and prognosis of 14 hub genes in EOC. GEPIA is based on 9,736 tumors and from cancer genomic map [The Cancer Genome Atlas (TCGA)] and genotype-tissue expression (Tang et al., 2017). We found eight key genes whose expression levels were consistent with the prognosis and was further validated in ONCOMINE database. (www.oncomine.org) (Rhodes et al., 2007) and The Human Protein Atlas (http://www.proteinatlas.org/) (Lindskog, 2015). Among them, five genes were associated with stage in our study based on GEPIA. Finally, ROC curve analysis was done to distinguish normal and cancer tissues.

### Gene Set Enrichment Analysis

In TCGA set validation, EOC samples were divided into two groups according to the median expression level. In order to identify potential function of the hub gene, we conducted a Gene set enrichment analysis (GSEA) (http://software.broadinstitute.org/gsea/index.jsp) (Subramanian et al., 2007) analysis to test whether a series of preferentially defined biological processes were enriched in the gene rank derived from DEGs between the two groups. In addition, we employed “clusterProfiler” package in R to handle the data of gene sets and use “Enrichplot” package to visualize the enriched pathways of the key genes. The adjusted *P* < 0.05 was set as the cutoff criterion.

## Result

### Identification of DEGs in EOC and the Enrichment of These Genes

**Figure 1:**
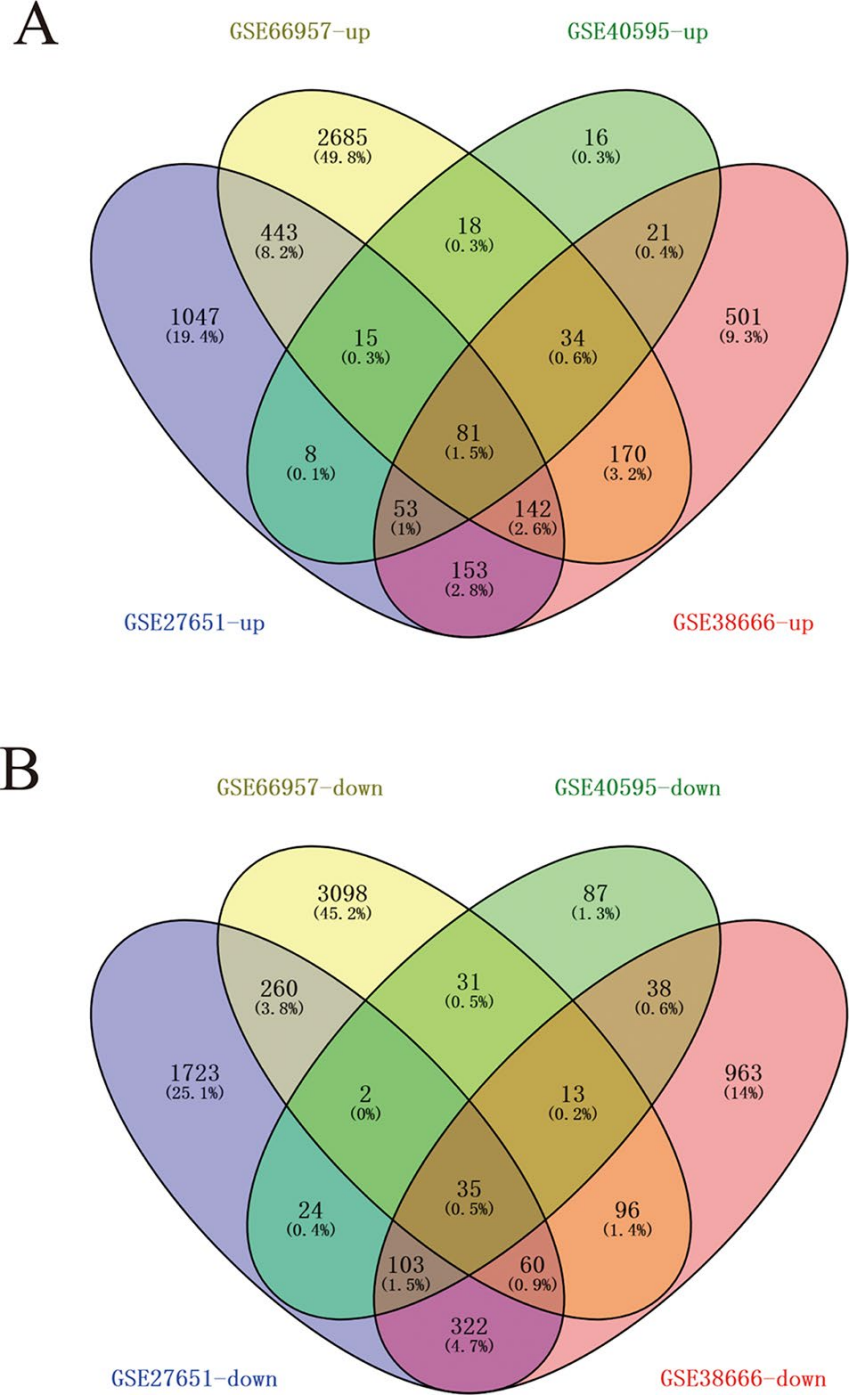
A total of 116 commonly changed differentially expressed genes (DEGs) which were divided into up- and down-regulated groups, were identified from four transcription profile datasets (GSE27651, GSE38666, GSE40595, and GSE66957) using online website. Calculate and draw custom Venn diagrams (available online: http://bioinformatics.psb.ugent.be/webtools/Venn/). Statistically significant DEGs were defined with adjusted *P* < 0.05 and |log2FC| > 1 as the cutoff criterion. **(A)** Up-regulated genes **(B)** Down-regulated genes.

**Figure 2:**
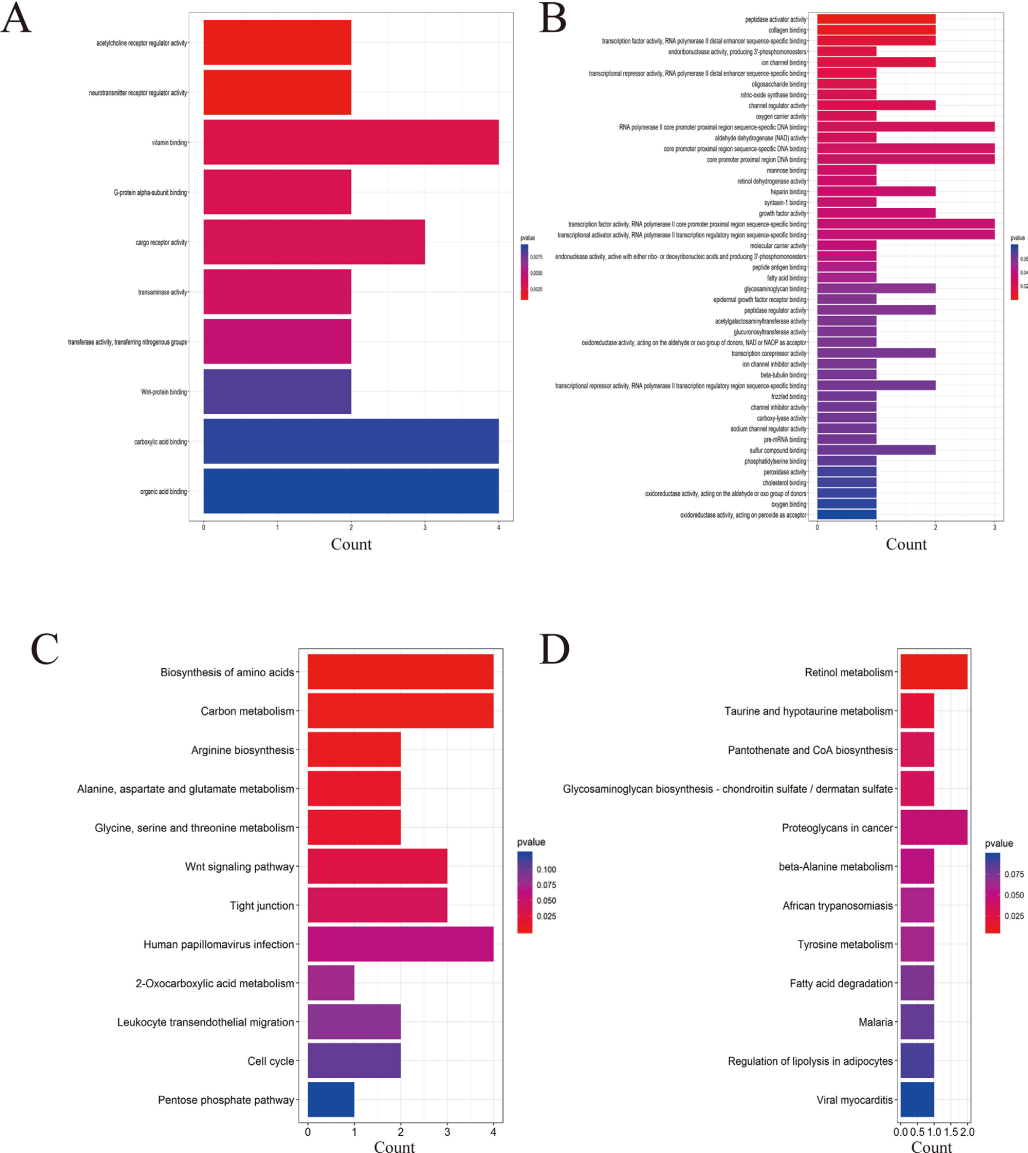
Gene Ontology (GO) and Kyoto Encyclopedia of Genes and Genomes (KEGG) analysis of the up- and downregulated DEGs. **(A)** GO analysis of upregulated genes associated with epithelial ovarian cancer (EOC). **(B)** GO analysis of downregulated genes associated with EOC. **(C)** KEGG analysis of upregulated genes associated with EOC. **(D)** KEGG analysis of downregulated genes associated with EOC.

Four data sets were obtained from the National Center for Biotechnology Information GEO database containing tumor tissue samples and normal ovarian tissue samples: GSE27651, GSE38666, GSE40595, and GSE66957. Then, the R package named “limma” was processed for analysis with adjusted *P* < 0.05 and |log2FC| >1. All DEGs were displayed in volcano maps (**Figure S1**). Top 200 genes in four databases were displayed in the heatmap (**Figure S2**). A total of 116 genes were finally obtained including 81 upregulated genes and 35 downregulated genes in the EOC tissue samples compared to the normal ovarian tissue samples (**Figure 1**). The data used to create **Figure 1** can be seen in **Table S1**. We also performed clusterProfiler package to do the functional analysis. In GO analysis, the hub upregulated genes were highly enriched in acetylcholine receptor regulator activity, neurotransmitter receptor regulator activity, and vitamin binding (**Figure 2A**); the hub downregulated genes were significantly enriched in peptidase activator activity, collagen binding and transcription factor activity, and RNA polymerase II distal enhancer sequence-specific binding (**Figure 2B**). The data used to create **Figure 2B** can be seen in **Table S2**. In KEGG analysis, the hub upregulated genes were significantly enriched in biosynthesis of amino acids and carbon metabolism (**Figure 2C**); the hub downregulated genes were significantly enriched in retinol metabolism (**Figure 2D**). The above research results can guide us to further study the significance of DEGs in EOC.

### GO and Pathway Enrichment Analysis of DEGs

**Figure 3:**
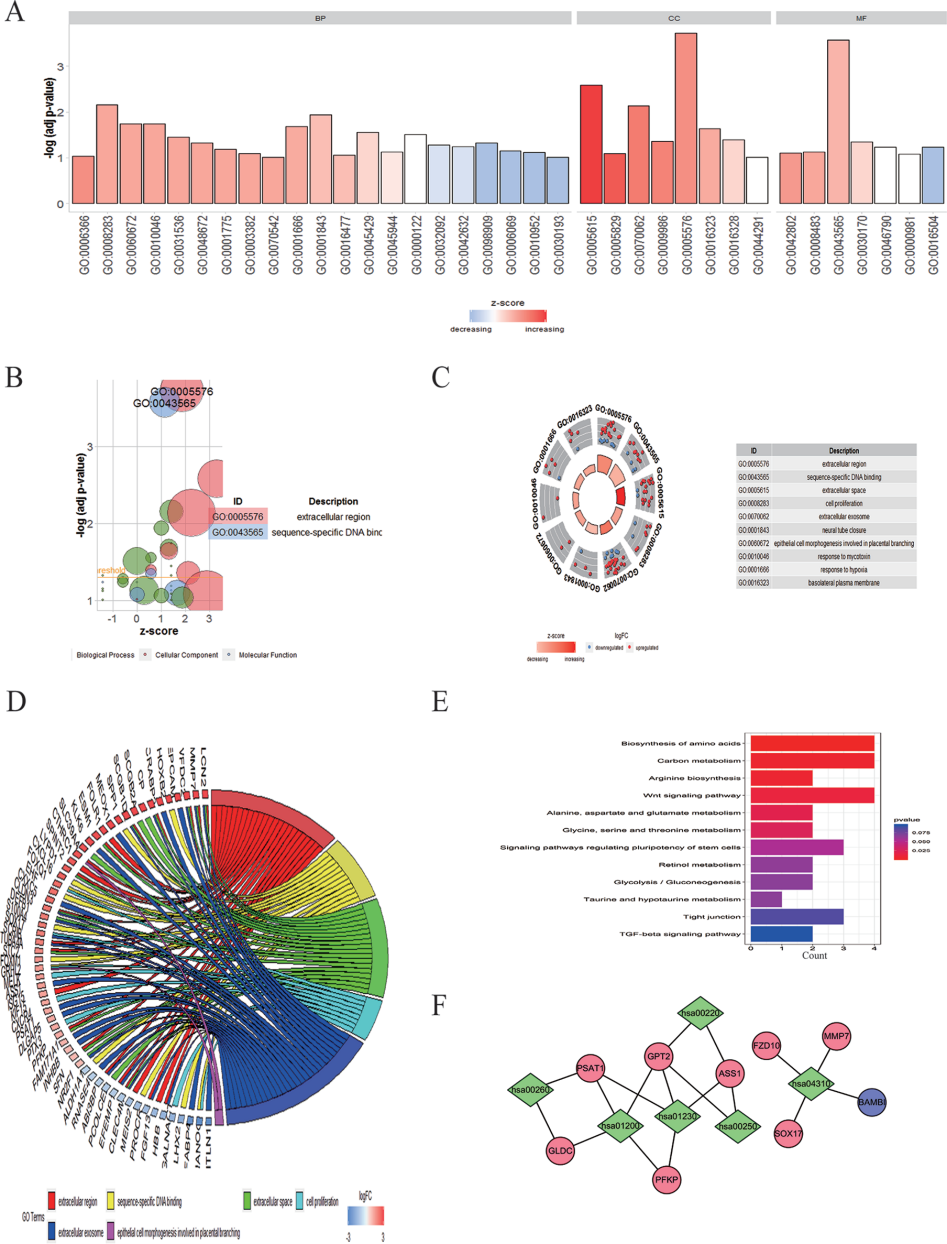
GO enrichment and KEGG analysis of DEGs in EOC. **(A)** GO analysis divided DEGs into three functional groups: molecular function, biological processes, and cell composition. **(B)** The bubble plot of enriched GO terms. The *z*-score is assigned to the *x*-axis, and the negative logarithm of the *P* value to the *y*-axis, as in the barplot (the higher the more significant). The size of the displayed circles is proportional to the number of genes assigned to the term. Green circles correspond to the biological process, red indicates the cellular component, and blue shows the molecular function category. **(C)** The top 10 GO terms of DEGs in EOC. The outer circle shows a scatter plot for each term of the logFC of the assigned genes. Red circles display upregulation and blue ones downregulation. **(D)** Distribution of DEGs in cervical cancer for different GO-enriched functions. **(E)** KEGG analysis of DEGs. **(F)** The pathway–protein network of DEGs.

To clarify the major functions of these DEGs, we first explored the associated biological processes and KEGG pathways. The top highly enriched GO terms were divided into three groups: biological process (BP), cellular component (CC), and molecular function (MF) (**Figure 3A**). The most enriched GO terms in biological process was “transcription from RNA polymerase II promoter” (*P* < 0.05), that in cellular component was “extracellular space” and “cell proliferation” (*P* < 0.05), and that in molecular function was “sequence-specific DNA binding” (*P* < 0.05) (**Figures 3B, D**). We further obtained 10 significantly enriched GO terms with a *P* < 0.05. The DEGs included in the top 10 GO terms were shown in the **Figure 3C**. All the GO terms were exhibited in **Table S4**.

In the KEGG analysis, the DEGs were mostly enriched in biosynthesis of amino acids, carbon metabolism, arginine biosynthesis, Wnt signaling pathway, alanine, aspartate, and glutamate metabolism, and glycine, serine, and threonine metabolism (**Figure 3E**).The pathway–protein network is shown in **Figure 3F**.

### Related Small Molecule Drugs Screening

**Figure 4:**
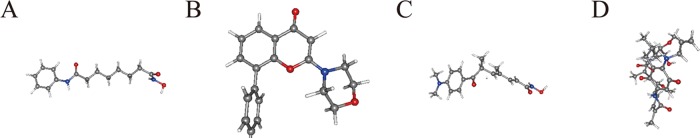
Three-dimensional diagram of the three most signiﬁcant drugs. **(A)** Vorinostat, **(B)** LY-294002, **(C)** trichostatin A, **(D)** tanespimycin.

We divided the 116 differentially expressed genes consisting of 35 downregulated genes and 81 upregulated genes into two groups, up- and downregulated, which were substituted into the CMap network tool. Among these highly significant correlated molecules, vorinostat, LY-294002, trichostatin A, and tanespimycin showed higher negative correlation with EOC. They all might have the potential therapeutic effects on EOC (**Table 1**). Three-dimensional structure of the top 4 candidate molecule drugs was found in Pubchem database and shown in **Figures 4A–D**.

**Table 1:** Results of ConnectivityMap (CMap) analysis.

Rank	CMap name	Mean	*N*	Enrichment	*P* value
1	vorinostat	−0.477	12	−0.639	0
2	LY-294002	−0.25	61	−0.393	0
3	trichostatin A	−0.359	182	−0.347	0
4	tanespimycin	−0.297	62	−0.307	0
5	folic acid	0.66	4	0.889	0.00014
6	gentamicin	0.613	4	0.886	0.00016
7	harmol	0.584	4	0.877	0.00034
8	amantadine	0.518	4	0.837	0.00109
9	trazodone	−0.63	4	−0.911	0.00124
10	hycanthone	0.449	4	0.823	0.00167

### PPI Network and Cluster Analysis

**Figure 5:**
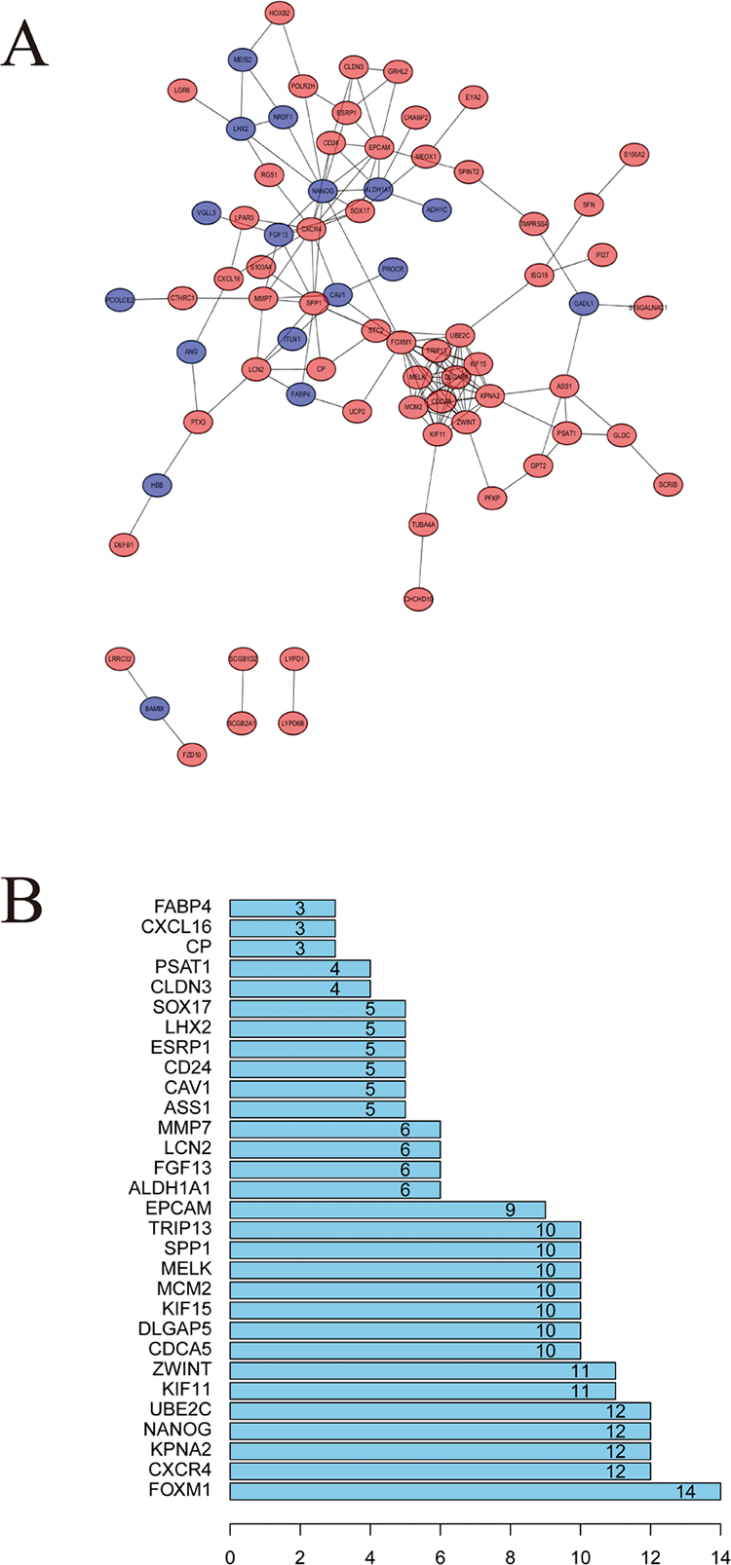
Protein–protein interaction (PPI) network analysis. **(A)** Using the STRING online database, a total of 161 DEGs were filtered into the DEGs PPI network. **(B)** Degree of the top 30 nodes in the PPI network.

**Figure 6:**
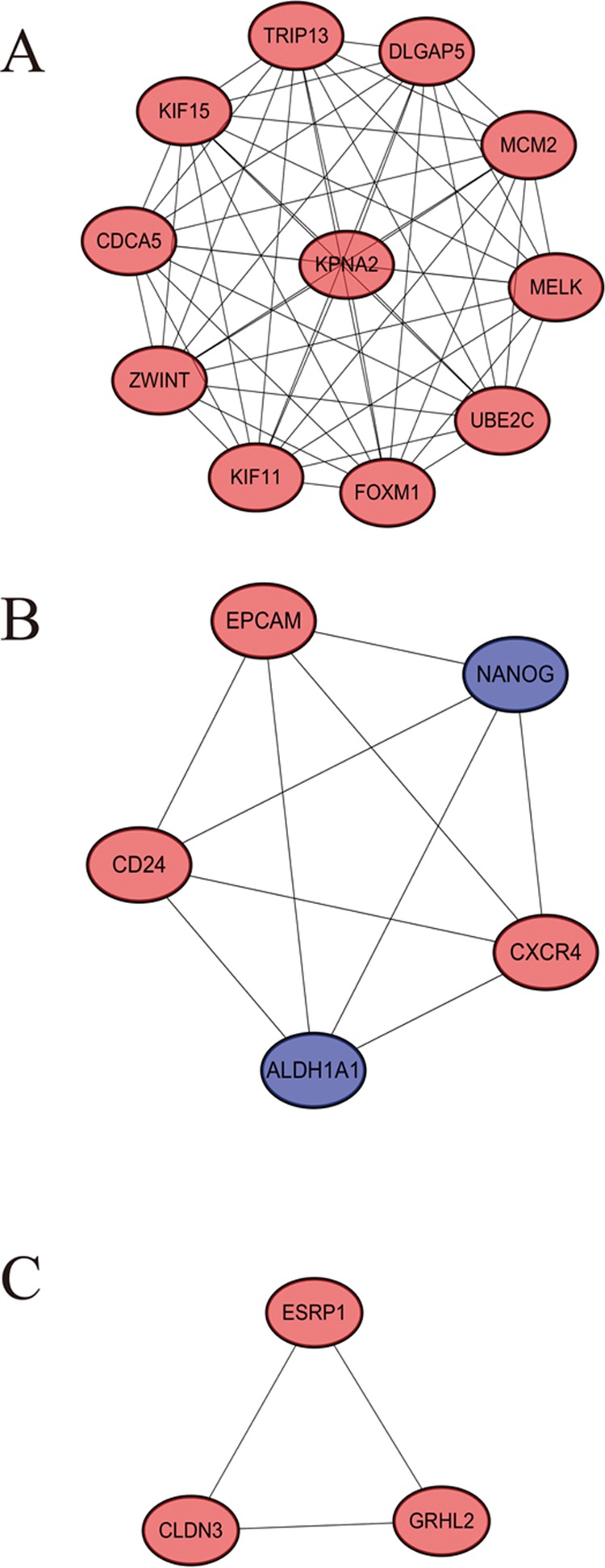
A signiﬁcant module selected from protein–protein interaction network. **(A)** Cluster 1, **(B)** cluster 2, **(C)** cluster 3.

STRING website screened 114 DEGs into PPI complex, which demonstrated 114 nodes and 157 edges (**Figure 5A**), and 30 important proteins were identified (**Figure 5B**). After that, we applied the MCODE, and three clusters were obtained. Among them, cluster 1 contained 11 core proteins and got the highest score in these clusters (**Figure 6A**), cluster 2 contained 5 proteins (**Figure 6B**), and cluster 3 contained 3 proteins (**Figure 6C**). These results may indicate that the 19 DEGs influence EOC.

**Figure 7:**
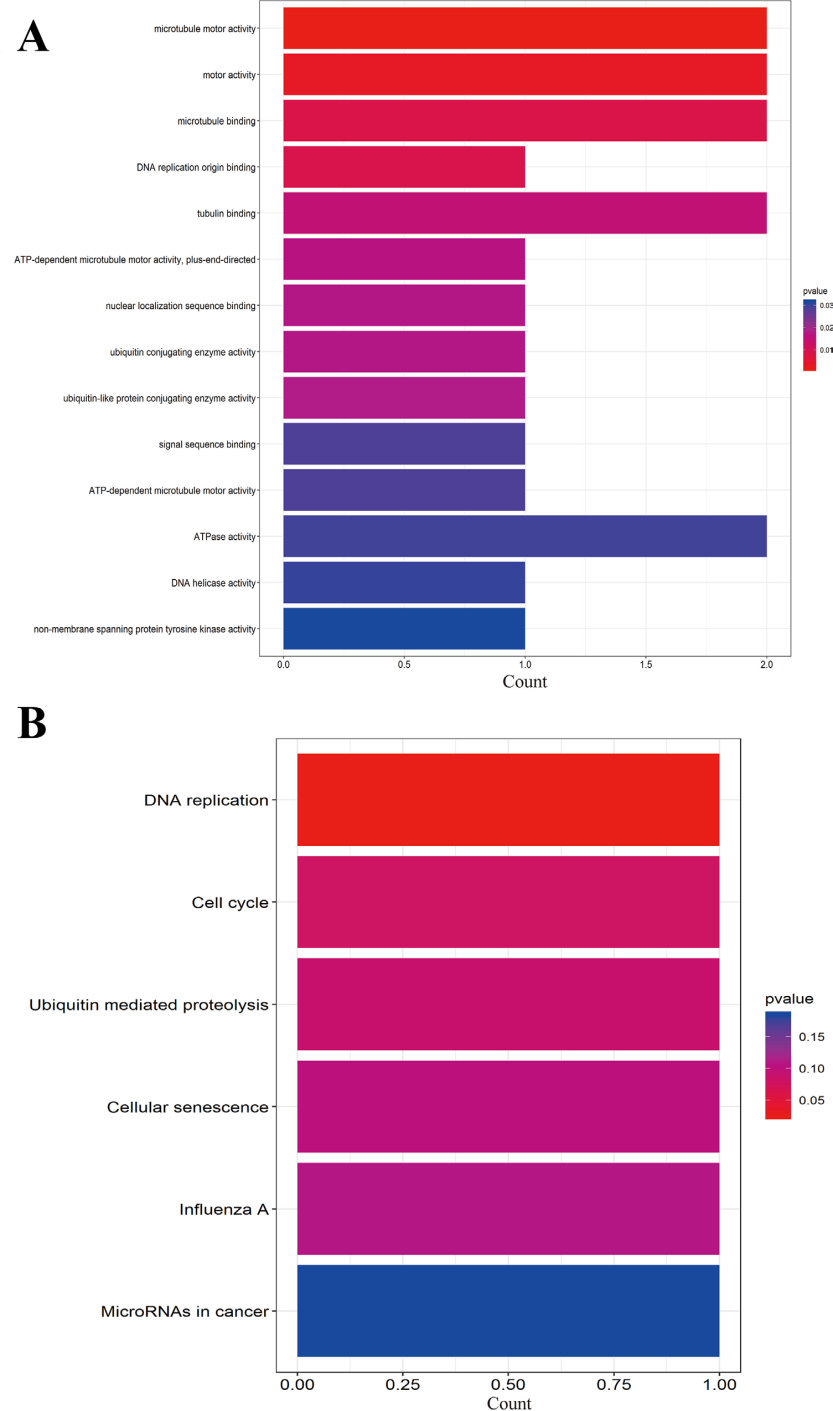
**(A)** GO analysis of these signiﬁcant molecule in cluster 1. **(B)** KEGG analysis of these signiﬁcant molecule in cluster 1.

We further performed the functional analysis of cluster 1. In GO analysis, the DEGs of cluster 1 were mostly enriched in microtubule motor activity, motor activity, and microtubule binding (**Figure 7A**). In KEGG analysis, the DEGs of cluster 1 were mostly enriched in DNA replication and cell cycle (**Figure 7B**). All pathways of signiﬁcant molecule in cluster 1 are shown in **Table S3**.

### Hub Gene Validation

**Figure 8:**
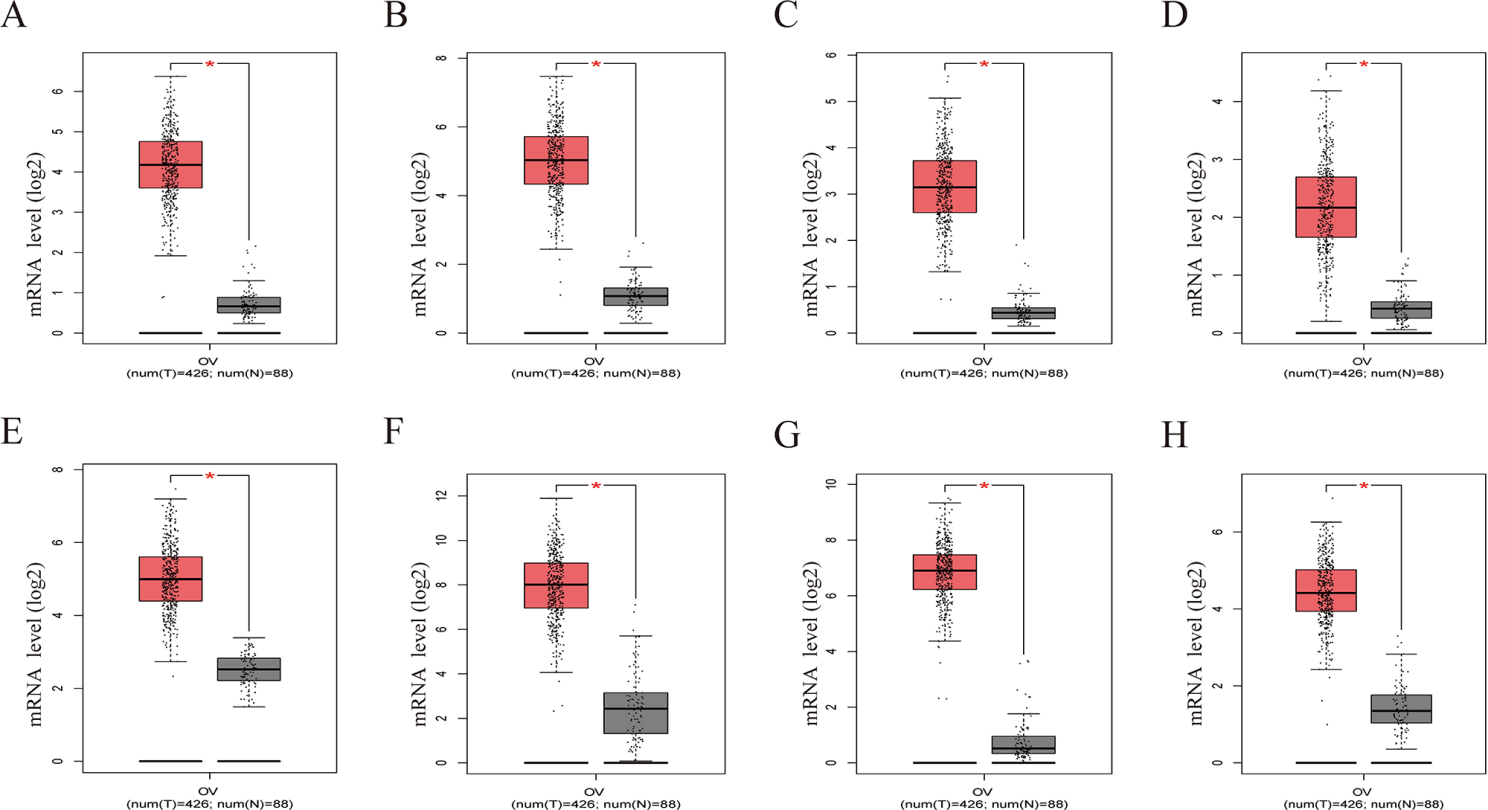
Gene Expression Profiling Interactive Analysis (GEPIA) was performed to validate higher expression of eight hub genes in EOC samples compared with normal samples. Red box was the cancer tissue group, gray was the normal tissue group, and asterisk represented *p* < 0.01. The dots represented expression in each sample. **(A)** CDCA5, **(B)** FOXM1, **(C)** KIF11, **(D)** KIF15, **(E)** MCM2, **(F)** SPP1, **(G)** UBE2C, **(H)** ZWINT.

**Figure 9:**
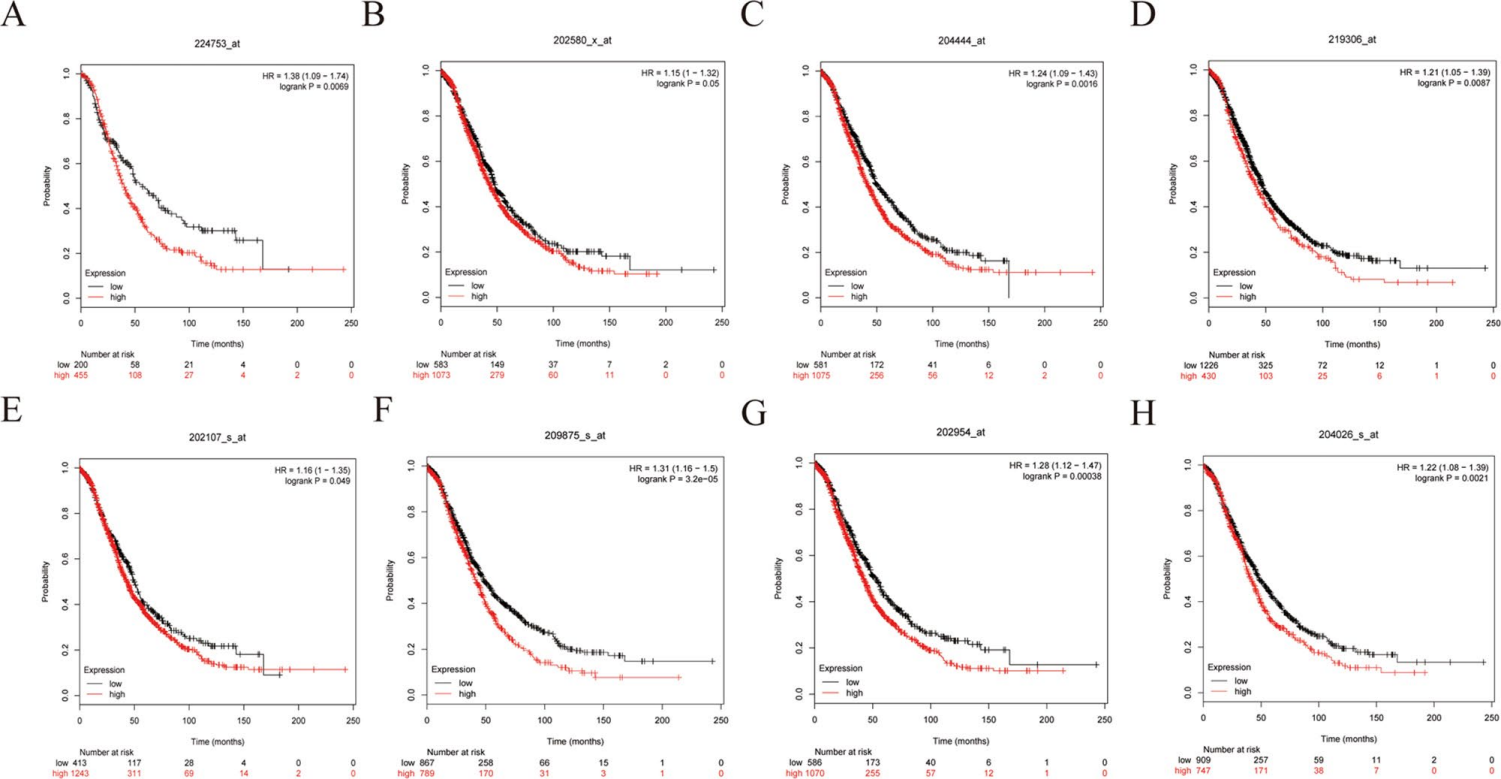
Overall survival (OS) curves of eight hub genes. **(A)** CDCA5, **(B)** FOXM1, **(C)** KIF11, **(D)** KIF15, **(E)** MCM2, **(F)** SPP1, **(G)** UBE2C, **(H)** ZWINT.

**Figure 10:**
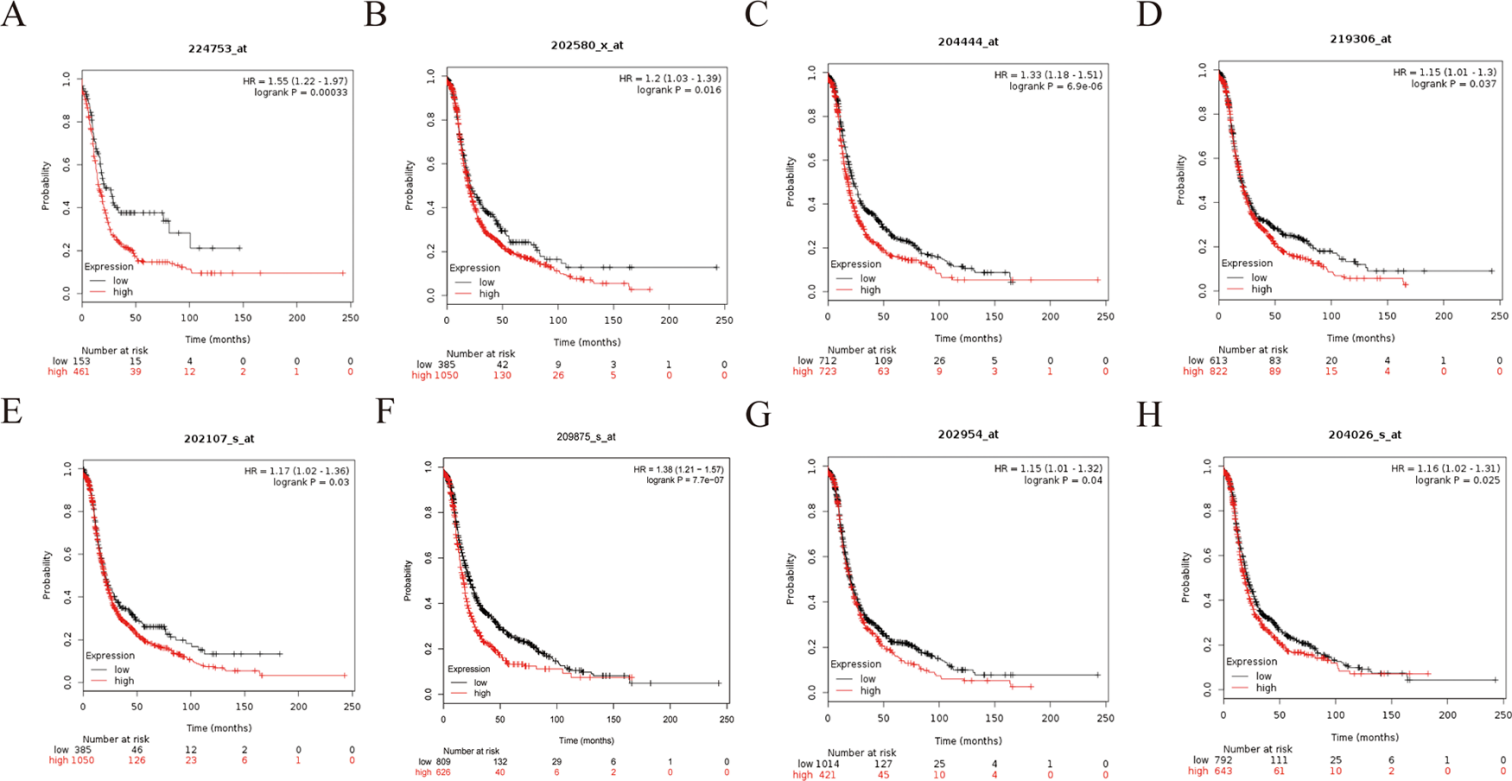
*Progression-free survival* (*PFS*) survival curves of eight hub genes. **(A)** CDCA5, **(B)** FOXM1, **(C)** KIF11, **(D)** KIF15, **(E)** MCM2, **(F)** SPP1, **(G)** UBE2C, **(H)** ZWINT.

**Figure 11:**
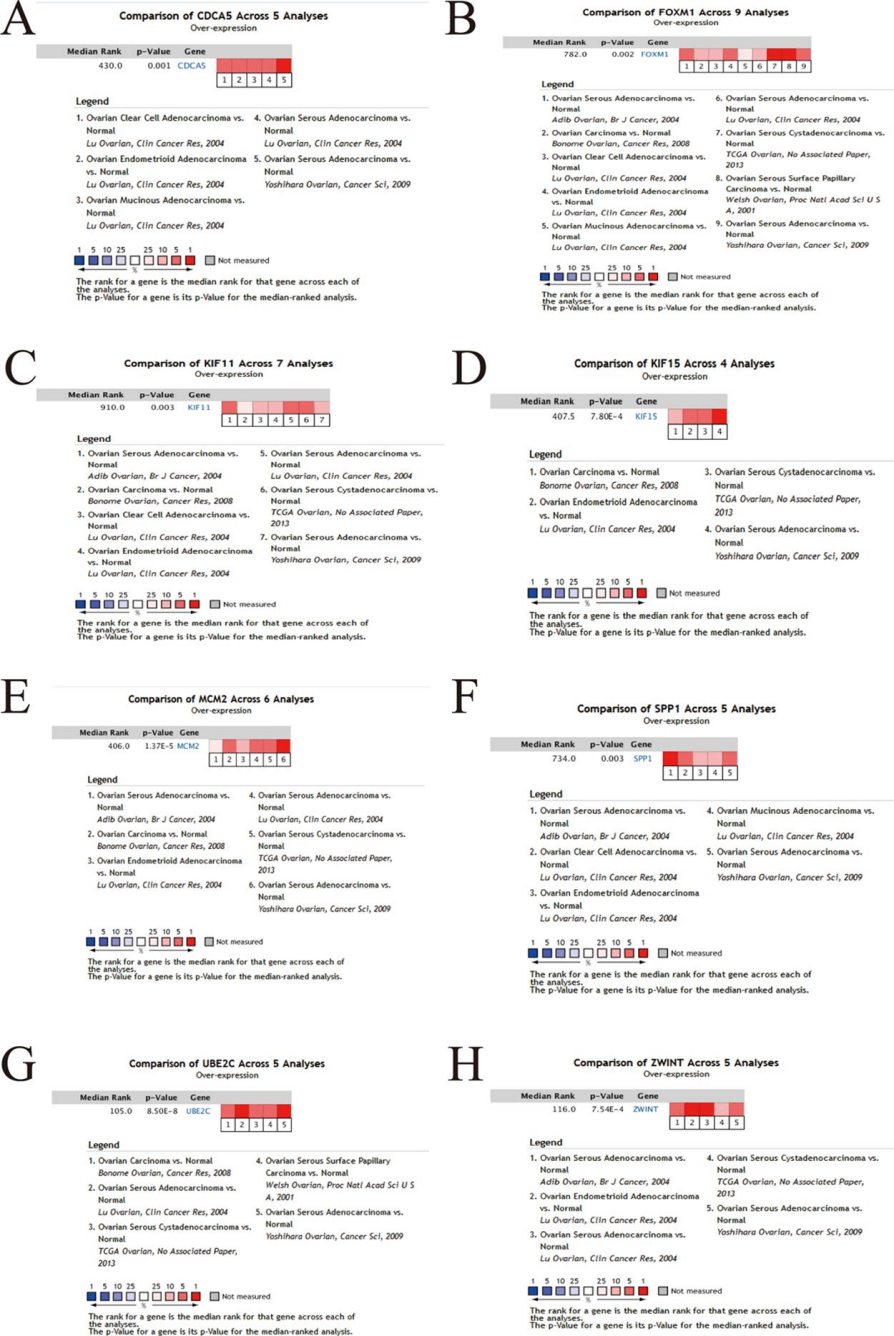
Eight hub genes expression within EOC across multiple datasets by Oncomine. **(A)** CDCA5, **(B)** FOXM1, **(C)** KIF11, **(D)** KIF15, **(E)** MCM2, **(F)** SPP1, **(G)** UBE2C, **(H)** ZWINT.

**Figure 12:**
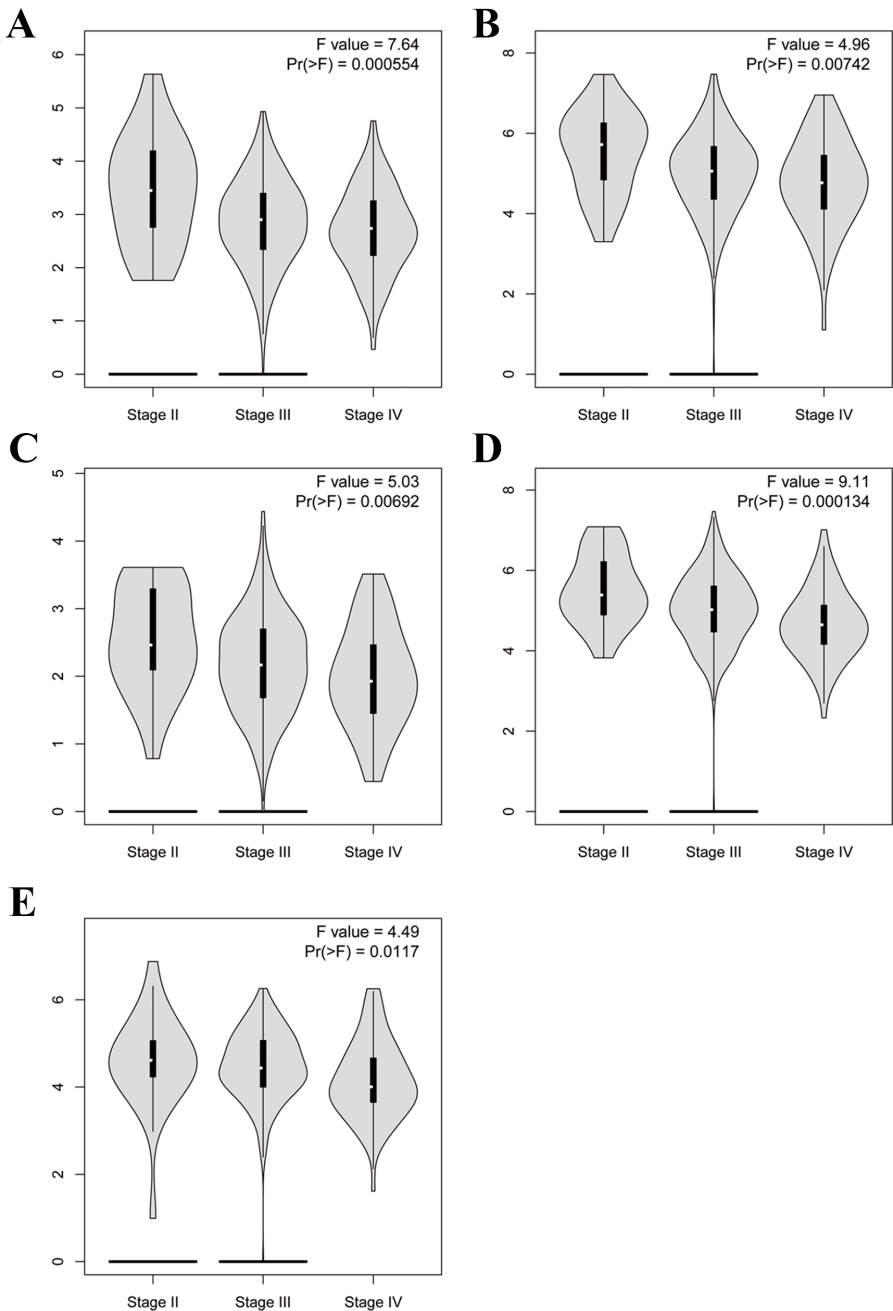
Violin plot showing five key genes expression in different major pathological stages of EOC. The *y*-axis represents log2 (TPM + 1). **(A)** CDCA5, **(B)** FOXM1, **(C)** KIF15, **(D)** MCM2, **(E)** ZWINT.

**Figure 13:**
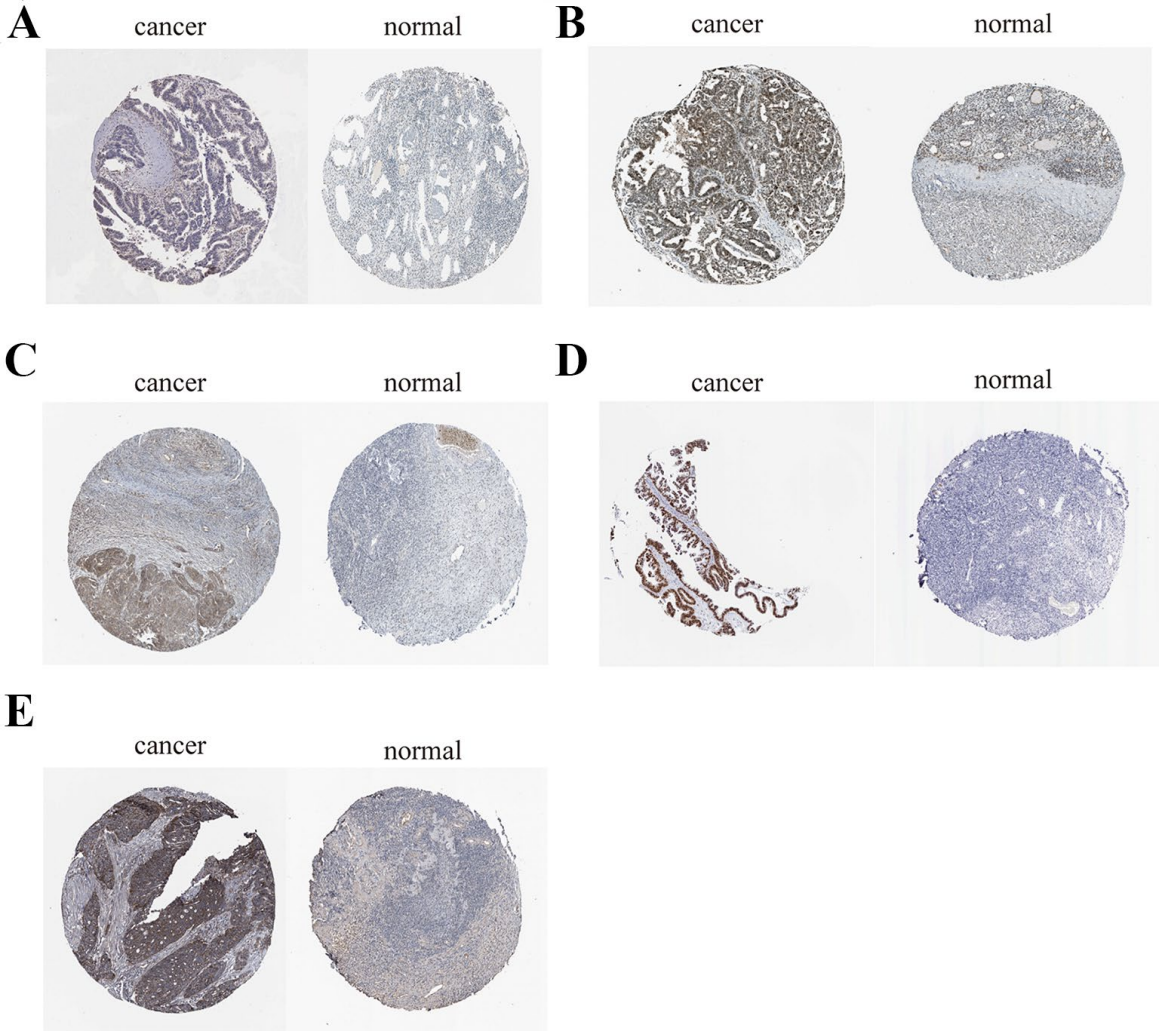
Immunohistochemistry of the five key genes based on the Human Protein Atlas. **(A)** Protein levels of CDCA5 in tumor tissue (staining: low; intensity: weak; quantity: > 75%). Protein levels of CDCA5 in normal tissue (staining: not detected; intensity: weak; quantity: < 25%). **(B)** Protein levels of FOXM1 in tumor tissue (staining: high; intensity: strong; quantity: > 75%). Protein levels of FOXM1 in normal tissue (staining: medium; intensity: moderate; quantity: 75–25%);. **(C)** Protein levels of KIF15 in tumor tissue (staining: medium; intensity: moderate; quantity: > 75%). Protein levels of KIF15 in normal tissue (staining: not detected; intensity: weak; quantity: < 25%). **(D)** Protein levels of MCM2 in tumor tissue (staining: high; intensity: strong; quantity: > 75%). Protein levels of MCM2 in normal tissue (staining: medium; intensity: moderate; quantity: 75–25%). **(E)** Protein levels of ZWINT in tumor tissue (staining: high; intensity: strong; quantity: > 75%). Protein levels of ZWINT in normal tissue (staining: low; intensity: weak; quantity: > 75%).

**Figure 14:**
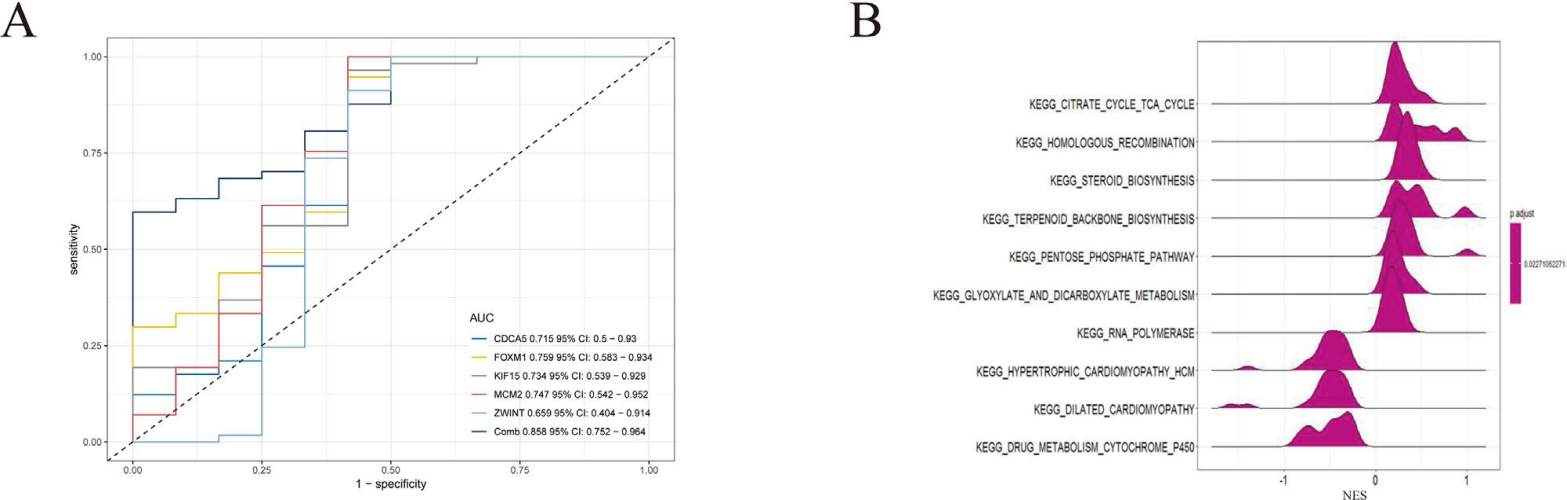
**(A)** Receiver operating characteristic (ROC) curve analysis and area under the curve (AUC) statistics were implemented on different databases to evaluate the capacity of key genes to distinguish EOC and normal tissues. **(B)** GSEA was applied to obtain biological process enriched in five key genes with highly expressed samples.

To further demonstrate the effect of these genes in EOC, we performed survival analysis on all the 114 DEGs first (**Table S5**), and 14 genes were obtained as candidate genes according to degree ≥10. Then, CDCA5, FOXM1, KIF11, KIF15, MCM2, SPP1, UBE2C, and ZWINT, which showed higher expression in CESC samples compared with normal samples (**Figure 8**), were negatively relative to overall survival of EOC patients (**Figure 9**). Patients with higher expression levels were worse than patients with lower expression levels. Similarly, overexpression of CDCA5, FOXM1, KIF11, KIF15, MCM2, SPP1, UBE2C, and ZWINT in tumors was significantly associated with *progression-free survival* in EOC patients (**Figure 10**). Expression analysis of cervical cancer versus normal performed on ONCOMINE also showed that expression of these eight genes were screened higher in EOC sample (**Figure 11**). Interestingly, we also found that five genes CDCA5, FOXM1, KIF15, MCM2, and ZWINT were relative to EOC stage by GEPIA analysis (**Figure 12**). In addition, we performed survival analysis based on stage I–II and stage III–IV. The results showed that the high expression of five hub genes was significantly worse than that of low expression in the stage I/II, but there was no statistical significance in stage III/IV (**Figure S3**). Immunohistochemistry also suggested that, compared with normal tissues, the protein expression level of these five genes were obviously higher in tumor tissues (**Figure 13**). In addition, ROC curve analysis was implemented to evaluate the capacity of hub genes to distinguish EOC and normal tissues in GES66957, CDCA5, FOXM1, KIF15 and MCM2, exhibiting better diagnostic efficiency for normal and tumor tissues, and the combined diagnosis of these five genes was more effective. The value of AUC was 0.858 (**Figure 14A**). However, efficiency of the ROC analysis between stage I–II and stage III–IV was weak (**Figure S4**). In addition, the univariate and multivariate Cox proportional hazards regression showed that the ZWINT was an independent prognostic indicator for overall survival among EOC patients (**Table 2**).

**Table 2:** Univariate and multivariate analyses of the correlation of CDCA5, FOXM1, KIF15, MCM2, and ZWINT expression with overall survival (OS) among epithelial ovarian cancer patients.

Variables	Univariate analysis			Multivariate analysis		
	HR	95%CI	*P*	HR	95%CI	*P*
Age (≤60 vs. >60)	1.358	1.038–1.775	**0.025**	1.275	0.944–1.722	0.113
Stage (stages I and II vs. stages III and IV)	1.095	0.647–1.852	0.735	0.938	0.542–1.622	0.819
Grade (G1 and G2 vs. G3 and G4)	1.373	0.904–2.084	0.137	1.185	0.752–1.868	0.464
CDCA5	0.991	0.965–1.018	0.517	1.003	0.960–1.048	0.888
FOXM1	1.002	0.992–1.013	0.648	1.006	0.990–1.021	0.440
KIF15	0.991	0.910–1.079	0.842	1.002	0.885–1.134	0.977
MCM2	0.998	0.988–1.008	0.675	1.002	0.990–1.014	0.692
ZWINT	0.982	0.965–0.999	**0.038**	0.977	0.956–0.998	**0.035**

Bold values indicate P < 0.05. HR, hazard ratio; CI, confidence interval.

### Gene Set Enrichment Analysis

To identify the potential function of these five genes in TCGA OV databases, GSEA was conducted to search KEGG pathways enriched in the highly expressed samples. As a result, 10 gene sets “citrate cycle tricarboxylic acid (TCA) cycle,” “homologous recombination,” “steroid biosynthesis,” “pentose phosphate pathway,” “glyoxylate and dicarboxylate metabolism,” “RNA polymerase,” “hypertrophic cardiomyopathy,” “dilated cardiomyopathy,” and “drug metabolism cytochrome P450” were enriched (**Figure 14B**) (adjusted *P* < 0.05).

## Discussion

Although surgery and other treatment methods have been improved, the treatment effect and prognosis of advanced ovarian cancer patients are very poor due to the difficulty in the diagnosis of ovarian cancer. Although many microlevel studies have been carried out, they are not yet mature. In this study, we integrated four gene chips from GEO databases and selected 116 DEGs between tumor and nontumor samples (81 expression levels were upregulated and 35 expression levels were downregulated), and further functional analysis was performed.

GO analysis displayed that the upregulated DEGs were mainly enriched in acetylcholine receptor regulator activity, and the downregulated genes were highly enriched in peptidase activator activity. The KEGG analysis showed that the upregulated DEGs were highly involved in biosynthesis of amino acids, while the hub downregulated genes were highly enriched in retinol metabolism.

Acetylcholine receptor regulator activity is often mentioned in lung cancer (Wang and Hu, 2018). Peptidase activator activity has been shown to be involved in the regulation of prostate cancer (Fuhrman-Luck et al., 2016). Biosynthesis of amino acids also has a relationship with the treatment of tumors (Manig et al., 2017). Retinol metabolism has been shown to be associated with breast cancer and gallbladder cancer (Chen et al., 1997). These key pathways are related to the occurrence and development of human tumors but have not been studied in detail in EOC, so functional analysis has certain guiding significance.

Several small molecules with potential therapeutic efﬁcacy against EOC were identified. Among them, the most relevant vorinostat, LY-294002, trichostatin A, and tanespimycin had been shown to have different degrees of association with tumors. Vorinostat is a small molecule inhibitor of both class I and II histone deacetylase enzymes (Munster et al., 2001) that functions by altering acetylation, affecting apoptotic pathways (Finnin et al., 1999). Vorinostat, which has been approved by the US Food and Drug Administration, is used to treat a variety of malignancies including ovarian cancer (Ma et al., 2017). LY-294002 has no clinical applications at present. Trichostatin A, as a histone deacetylase inhibitor, has been shown to exhibit anticancer effects in combination with radiotherapy or chemotherapy (Ranganathan and Rangnekar, 2005; Hajji et al., 2008). In the early 1990s, there were experiments that demonstrated that tanespimycin had antitumor activity against various human-derived tumor cell lines (Erlichman, 2009). The above three drugs have been identified to have antitumor effect in the past. The PPI network analyzed DEGs and displayed 114 nodes. The MCODE plug-in filtered out three related modules. The correlation of module 1 was the most significant. We performed survival analysis on 11 genes which belong to module 1 and found that patients with these DEG disorders had a poor prognosis. Among these genes, CDCA5, FOXM1, KIF15, MCM2, and ZWINT were the most reported genes associated with cancer progression, including EOC. ROC curve analysis demonstrated these genes had better diagnostic efficiency for normal and tumor tissues, and the combination of diagnosis was more effective. Meanwhile, the univariate and multivariate Cox proportional hazards regression showed that ZWINT was an independent prognostic indictor among EOC patients. Besides, GSEA suggested that the five genes were mostly enriched in citrate cycle TCA cycle, homologous recombination, and steroid biosynthesis.

Interestingly, the study by Ren JG et al. found that citrate suppressed tumor growth through inhibition of glycolysis, the TCA cycle and the insulin-like growth factor-1 receptor pathway (Ren et al., 2017). Homologous recombination deficiency was closely related to ovarian cancer and breast cancer (Zhao et al., 2017; Da et al., 2018). These examples show that the results of GESA analysis can be used as a reference for oncogenesis studies to some extent.

Cell-division cycle-associated 5 (CDCA5), also known as sororin, is thought to play a critical role in ensuring the accurate separation of sister chromatids during the S and G2/M phases of the cell cycle through interactions with cohesin and cdk1 (Schmitz et al., 2007; Borton et al., 2016). CDCA5 has also been shown to interact with ERK as well as cyclin E1, a critical regulator of the G1/Smitotic checkpoint (Schmitz et al., 2007; Nguyen et al., 2010; Borton et al., 2016). Recent studies have correlated the expression of CDCA5 with tumorigenesis and tissue invasion in several cancers, including oral squamous cell cancer, nonsmall cell lung cancer, urothelial cell carcinoma, and gastric cancer (Chang et al., 2015; Tokuzen et al., 2016). However, the gene has not been reported in ovarian cancer and deserves further study.

FOXM1 is a member of the forkhead box (Fox) transcription factor family, which is known as an oncogene involved in breast cancer, cervix cancer, prostate cancer, and so on. In agreement with previously published studies (Lok et al., 2011; Wen et al., 2014; Zhao et al., 2014; Zhou et al., 2014; Chiu et al., 2015), our experimental findings demonstrated that FOXM1 was overexpressed in EOC and negatively associated with prognosis of EOC patients.

KIF15 is the breast cancer tumor antigen and is necessary for the maintenance of spindle bipolarity (Scanlan et al., 2001). KIF15 supports K5I resistance in HeLa cells (Sturgill et al., 2016), which is shown to act as target for endocrine therapy-resistant breast cancer (Zou et al., 2014). The same result existed in lung adenocarcinoma and may play a vital role in regulating the cell cycle (Bidkhori et al., 2013). Our study reported for the first time that KIF15 expressed higher in EOC and led to the bad outcome of EOC patients.

Minichromosome maintenance (MCM) 2 is one of six related proteins that comprise the MCM complex (MCM2-7), which has an essential role in DNA replication (Bochman and Schwacha, 2008). Previous studies using human samples have established MCM2 as a proliferation marker of cancer cells. High expression of MCM2 level in malignant tumors, including ovarian cancer, is associated with several clinicopathological parameters such as advanced tumor grade, advanced stage, and poor prognosis (Davies et al., 2002; Going et al., 2002; Dudderidge et al., 2005; Majid et al., 2010; Abe et al., 2015). In our study, we also found that MCM, which had higher expression in EOC, was relative to bad outcome of EOC patient.

ZWINT belongs to the kinetochore complex and is a protein that interacts with ZW10 and participates in chromosome movement (Woo et al., 2015). Endo et al. found that ZWINT promoted cell growth, and targeting KWINT inhibited breast cancer cell growth (Endo et al., 2012). There is also a bioinformation research that reported this gene in OC, which is similar to ours. In summary, CDCA5, FOXM1, KIF15, MCM2, and ZWINT was involved in cell mitosis and supported our research results by affecting the cell cycle regulation of tumor pathogenesis.

There are several limitations in our study as follows. First, there is an urgent need for biological experiments to validate our results because our research is based on data analysis. Second, we lack the molecular mechanisms for these genes, and we will incorporate these for further exploration. In the future, we will further design experiments (including PCR, Western blot, immunohistochemistry, etc.) based on specific mechanisms, conduct in-depth research, and improve the inadequacies.

## Conclusion

In our study, we adopted 4 GEO chips to demonstrate 116 DEGs. Then, we comprehensively analyzed GEPIA, ONCOMINE, and other databases. We identified that CDCA5, FOXM1, KIF15, MCM2, and ZWINT were related to EOC. At the same time, our study also analyzed the potential new drugs for the treatment of ovarian cancer based on the DEGs. In a word, our research proved that bioinformatics analysis might open up new directions for cancer research. More therapeutic targets will be tapped if further clinical trials are combined.

## Data Availability Statement

The datasets in this study are available from GEO database (https://www.ncbi.nlm.nih.gov/geo/) using accessions numbers GSE27651, GSE38666, GSE40595 and GSE66957 and TCGA database (https://cancergenome.nih.gov/).

## Author Contributions

WC and JL designed the project. JL, HM, and SL contributed on data analysis and prepared the main manuscript. All authors reviewed the manuscript.

## Funding

This work was supported by the National Natural Science Foundation of China (81872119 and 81472442) and the Jiangsu province medical innovation team (CXTDA2017008).

## Conflict of Interest

The authors declare that the research was conducted in the absence of any commercial or financial relationships that could be construed as a potential conflict of interest.

## Supplementary Material

Supplementary Figure 1 Volcano map of DEGs on the basis of |fold change| > 1 and a corrected P-value <0.05. (A) GSE27651 (B) GSE38666 © GSE40595 (D) GSE66957. Red dots represented the upregulated DEGs. Green dots represented the down-regulated DEGs. Black dots represented noDEGs.

Supplementary Figure 2 Hierarchical clustering heatmap of top 200 DEGs. (A) GSE27651 (B) GSE38666 © GSE40595 (D) GSE66957. Red indicates that the expression of genes is relatively upregulated, green indicates that the expression of genes is relatively downregulated, and black indicates no significant changes in gene expression.

Supplementary Figure 3 The survival analysis based on stage I-II and stage III-IV. The one on the left in the figure was survival analysis based on stage I-II, the right one was stage III-IV.

Supplementary Figure 4 ROC analysis between stage I-II and stage III-IV.
