# Supplementary figures and images for: Identification of Potential Biomarkers in Association With Progression and Prognosis in Epithelial Ovarian Cancer by Integrated Bioinformatics Analysis
Source: Front Genet. 2019 Oct 24;10:1031. doi: 10.3389/fgene.2019.01031 (PMC6822059; doi:10.3389/fgene.2019.01031)

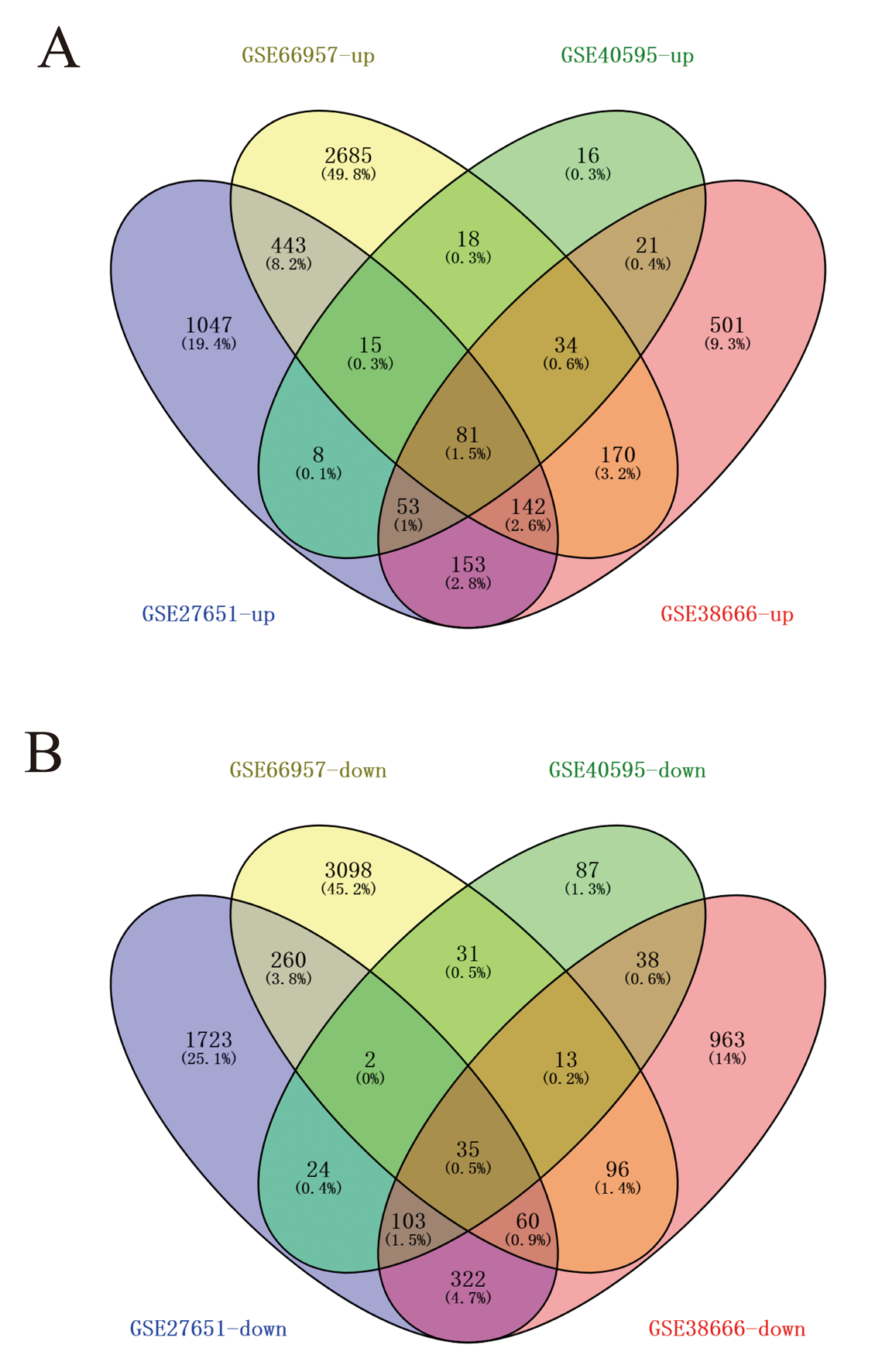

Supplement: Supplementary Figure 1 — Volcano map of DEGs on the basis of |fold change| > 1 and a corrected P-value <0.05. (A) GSE27651 (B) GSE38666 © GSE40595 (D) GSE66957. Red dots represented the upregulated DEGs. Green dots represented the down-regulated DEGs. Black dots represented noDEGs. [file Image_1.tif]

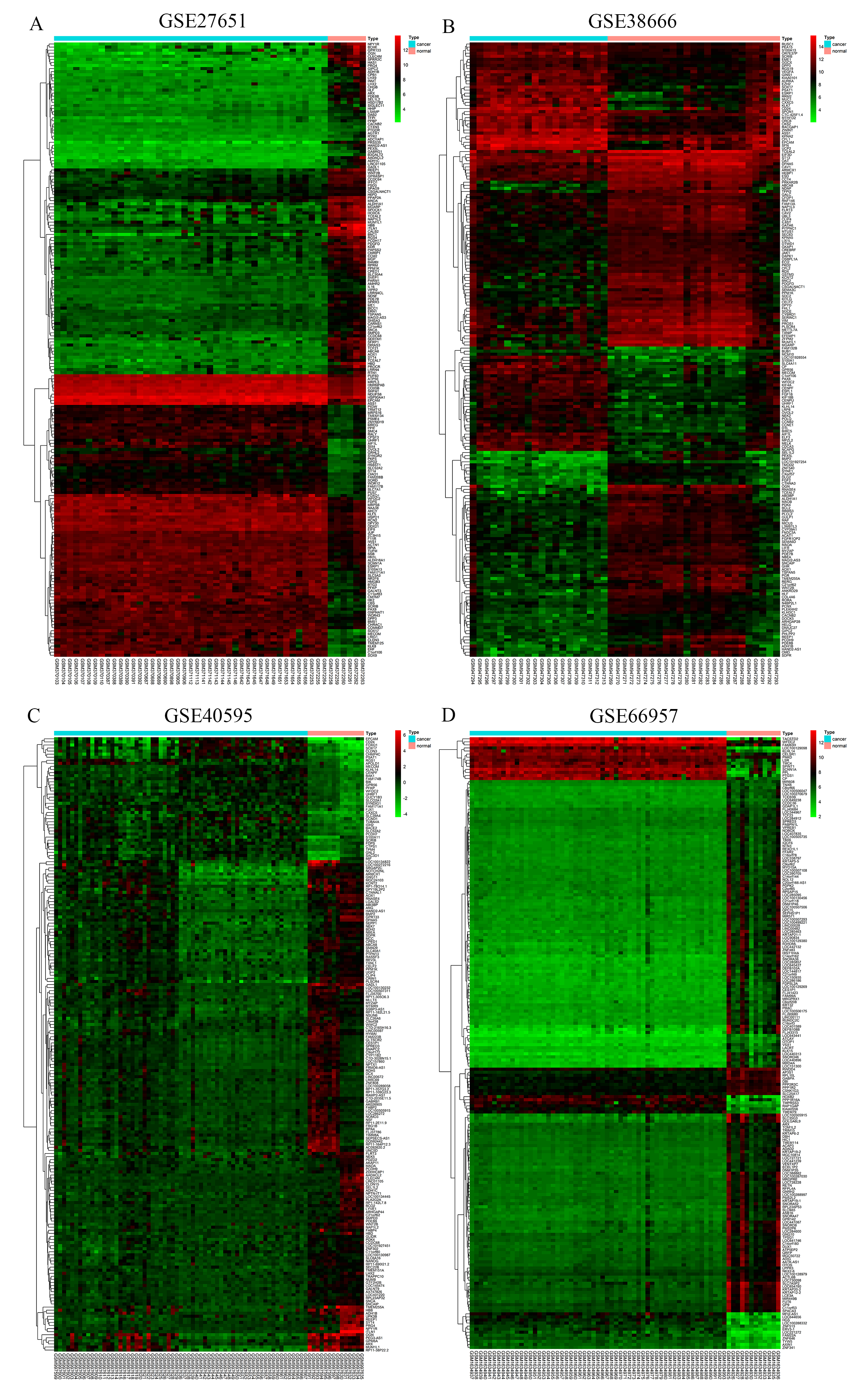

Supplement: Supplementary Figure 2 — Hierarchical clustering heatmap of top 200 DEGs. (A) GSE27651 (B) GSE38666 © GSE40595 (D) GSE66957. Red indicates that the expression of genes is relatively upregulated, green indicates that the expression of genes is relatively downregulated, and black indicates no significant changes in gene expression. [file Image_2.tif]

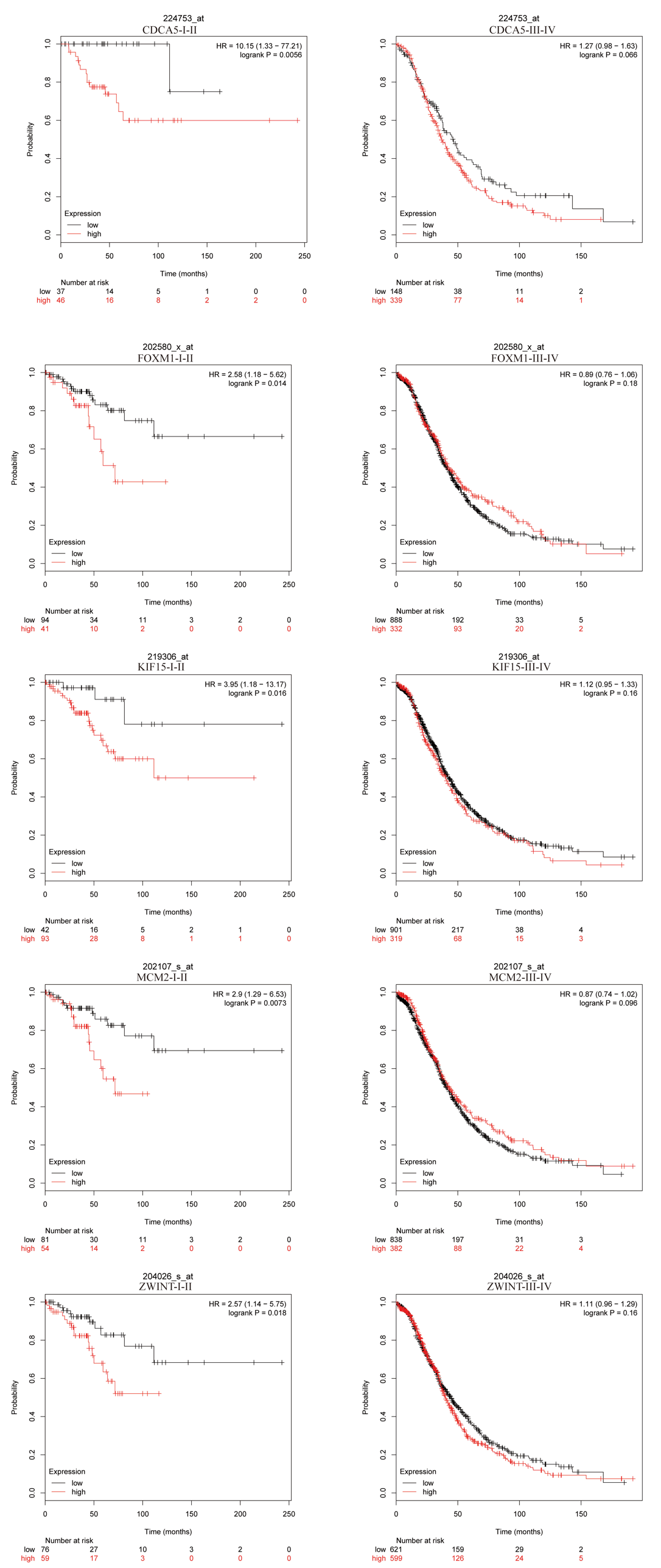

Supplement: Supplementary Figure 3 — The survival analysis based on stage I-II and stage III-IV. The one on the left in the figure was survival analysis based on stage I-II, the right one was stage III-IV. [file Image_3.jpeg]

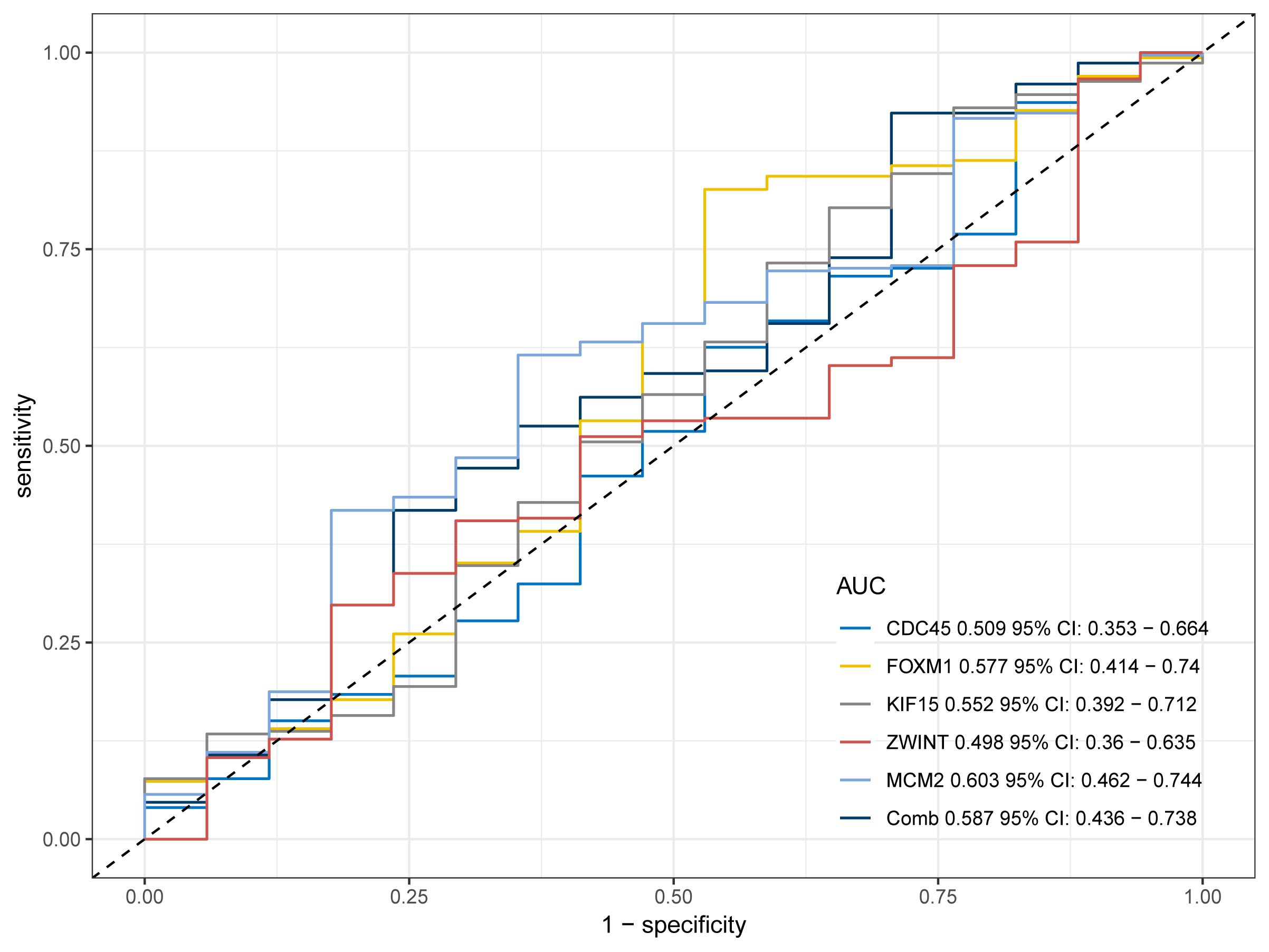

Supplement: Supplementary Figure 4 — ROC analysis between stage I-II and stage III-IV. [file Image_4.tif]
