# Supplementary material for: Identification of Potential Biomarkers in Association With Progression and Prognosis in Epithelial Ovarian Cancer by Integrated Bioinformatics Analysis
Source: Front Genet. 2019 Oct 24;10:1031. doi: 10.3389/fgene.2019.01031 (PMC6822059; doi:10.3389/fgene.2019.01031)
Supplement: Supplementary file 5 [file Table_1.docx]

**Supplementary Table 1：LogFC of 116 DEGs**

| **Gene** | **logFC** |
| --- | --- |
| ESRP1 | 3.28823519 |
| C1orf106 | 1.859148762 |
| EPCAM | 5.056340302 |
| SOX17 | 2.133996882 |
| PFKP | 1.151487942 |
| MECOM | 1.063729234 |
| WFDC2 | 5.147608745 |
| SCRIB | 2.079952227 |
| FAM171A1 | 1.051972997 |
| FOXQ1 | 2.6439543 |
| CLDN3 | 2.35478703 |
| GRHL2 | 1.572093536 |
| ASS1 | 1.501075448 |
| EHF | 2.438034145 |
| TUBA4A | 2.058180497 |
| CP | 4.874761524 |
| CD24 | 4.434696861 |
| MCM2 | 1.826102197 |
| POLR2H | 1.074785088 |
| CXXC5 | 2.366728035 |
| UCP2 | 3.284188585 |
| CRABP2 | 4.976144152 |
| FZD10 | 3.679451312 |
| KLHL14 | 5.17659744 |
| PSAT1 | 1.334585242 |
| LYPD1 | 2.737085775 |
| SCGB2A1 | 4.514309179 |
| KLK6 | 2.922546844 |
| FOLR1 | 3.697136129 |
| BACE2 | 2.106844359 |
| LYPD6B | 4.150138651 |
| SLC39A4 | 2.916203074 |
| SPINT2 | 2.83221351 |
| MMP7 | 5.920591352 |
| RGS1 | 3.060873051 |
| EPHX4 | 2.135254219 |
| INHBB | 1.037893892 |
| ST6GALNAC1 | 4.129901125 |
| S100A2 | 1.84876021 |
| TRIP13 | 1.959574396 |
| TLCD1 | 3.058931792 |
| SFN | 1.030442209 |
| LCN2 | 6.407284781 |
| DEFB1 | 2.295512239 |
| KIF11 | 1.074563042 |
| ASRGL1 | 2.904787091 |
| LGR6 | 4.923334114 |
| TNNT1 | 4.069388461 |
| CHCHD10 | 1.26810675 |
| KPNA2 | 1.948096581 |
| PTX3 | 1.29061248 |
| ZWINT | 1.408531043 |
| LRRC32 | 2.678893165 |
| CXCR4 | 1.351495852 |
| EYA2 | 4.707146864 |
| SPP1 | 4.126190232 |
| SCGB1D2 | 4.133456762 |
| LPAR3 | 2.904286458 |
| TMPRSS4 | 3.415086905 |
| HIST1H1C | 1.739974267 |
| GPT2 | 1.388259455 |
| MELK | 1.555719655 |
| STC2 | 1.83135418 |
| LINC00284 | 1.148919577 |
| IFI27 | 2.703200022 |
| CDCA5 | 2.01939969 |
| MEOX1 | 3.894588525 |
| GLDC | 1.650052639 |
| SLC4A11 | 2.933407363 |
| DLGAP5 | 1.319549481 |
| KIF15 | 1.390955057 |
| GPR160 | 2.263797773 |
| UBE2C | 1.693710827 |
| CXCL16 | 2.688200621 |
| CTHRC1 | 2.866157149 |
| S100A4 | 2.147831354 |
| ESM1 | 3.470638203 |
| MPZL2 | 3.770879773 |
| HOXB2 | 5.007746496 |
| FOXM1 | 1.689332656 |
| ISG15 | 1.452045502 |
| ITLN1 | -3.116921832 |
| ARX | -1.608908604 |
| CLEC4M | -1.429322766 |
| HBB | -1.768293717 |
| SEL1L2 | -1.600272363 |
| SYT4 | -2.760177303 |
| ADH1C | -1.103907076 |
| GADL1 | -3.262335173 |
| CNRIP1 | -1.29519126 |
| LHX2 | -1.985728576 |
| PROCR | -1.555335226 |
| C21orf62 | -1.669528825 |
| CSGALNACT1 | -1.829190447 |
| BAMBI | -1.404263144 |
| CCDC68 | -1.347358545 |
| ALDH1A1 | -1.052275313 |
| FGF13 | -1.690669143 |
| SNCAIP | -1.338675891 |
| TMEM150C | -1.511937859 |
| RNASE4 | -1.071882972 |
| EFEMP1 | -1.202244214 |
| PCOLCE2 | -1.155563446 |
| PCDH9 | -1.459798729 |
| MYZAP | -1.700287377 |
| FABP4 | -2.837460261 |
| PEG3 | -1.484242316 |
| CELF2 | -1.432853525 |
| VGLL3 | -2.366554755 |
| MEIS2 | -1.528426515 |
| NANOG | -2.91070662 |
| ABI3BP | -1.146112395 |
| LOC401220 | -1.962917632 |
| CAV1 | -1.906708207 |
| RUNDC3B | -2.068456712 |
| NR2F1 | -1.019759401 |

DEGs, differentially expressed genes
