# Supplementary material for: Identification of Potential Biomarkers in Association With Progression and Prognosis in Epithelial Ovarian Cancer by Integrated Bioinformatics Analysis
Source: Front Genet. 2019 Oct 24;10:1031. doi: 10.3389/fgene.2019.01031 (PMC6822059; doi:10.3389/fgene.2019.01031)
Supplement: Supplementary file 6 [file Table_2.docx]

**Supplementary Table 2:** **GO analysis of downregulated genes associated with EOC**

| **ID** | **Term** | **Count** | **p-value** |
| --- | --- | --- | --- |
| GO:0016504 | peptidase activator activity | 2 | 0.001978179 |
| GO:0005518 | collagen binding | 2 | 0.005989113 |
| GO:0003705 | transcription factor activity, RNA polymerase II distal enhancer sequence-specific binding | 2 | 0.01345235 |
| GO:0016892 | endoribonuclease activity, producing 3'-phosphomonoesters | 1 | 0.017724964 |
| GO:0044325 | ion channel binding | 2 | 0.017859669 |
| GO:0001206 | transcriptional repressor activity, RNA polymerase II distal enhancer sequence-specific binding | 1 | 0.019480645 |
| GO:0070492 | oligosaccharide binding | 1 | 0.021233289 |
| GO:0050998 | nitric-oxide synthase binding | 1 | 0.022982901 |
| GO:0016247 | channel regulator activity | 2 | 0.023119826 |
| GO:0005344 | oxygen carrier activity | 1 | 0.024729486 |
| GO:0000978 | RNA polymerase II core promoter proximal region sequence-specific DNA binding | 3 | 0.026093693 |
| GO:0004029 | aldehyde dehydrogenase (NAD) activity | 1 | 0.026473049 |
| GO:0000987 | core promoter proximal region sequence-specific DNA binding | 3 | 0.028970266 |
| GO:0001159 | core promoter proximal region DNA binding | 3 | 0.029565205 |
| GO:0005537 | mannose binding | 1 | 0.02995113 |
| GO:0004745 | retinol dehydrogenase activity | 1 | 0.031685658 |
| GO:0008201 | heparin binding | 2 | 0.032983385 |
| GO:0017075 | syntaxin-1 binding | 1 | 0.033417185 |
| GO:0008083 | growth factor activity | 2 | 0.033741637 |
| GO:0000982 | transcription factor activity, RNA polymerase II core promoter proximal region sequence-specific binding | 3 | 0.033912388 |
| GO:0001228 | transcriptional activator activity, RNA polymerase II transcription regulatory region sequence-specific binding | 3 | 0.03631652 |
| GO:0140104 | molecular carrier activity | 1 | 0.036871253 |
| GO:0016894 | endonuclease activity, active with either ribo- or deoxyribonucleic acids and producing 3'-phosphomonoesters | 1 | 0.038593805 |
| GO:0042605 | peptide antigen binding | 1 | 0.045454247 |
| GO:0005504 | fatty acid binding | 1 | 0.04716194 |
| GO:0005539 | glycosaminoglycan binding | 2 | 0.055247467 |
| GO:0005154 | epidermal growth factor receptor binding | 1 | 0.057346185 |
| GO:0061134 | peptidase regulator activity | 2 | 0.057563218 |
| GO:0008376 | acetylgalactosaminyltransferase activity | 1 | 0.059033286 |
| GO:0015020 | glucuronosyltransferase activity | 1 | 0.059033286 |
| GO:0016620 | oxidoreductase activity, acting on the aldehyde or oxo group of donors, NAD or NADP as acceptor | 1 | 0.059033286 |
| GO:0003714 | transcription corepressor activity | 2 | 0.059912842 |
| GO:0008200 | ion channel inhibitor activity | 1 | 0.060717464 |
| GO:0048487 | beta-tubulin binding | 1 | 0.060717464 |
| GO:0001227 | transcriptional repressor activity, RNA polymerase II transcription regulatory region sequence-specific binding | 2 | 0.061338566 |
| GO:0005109 | frizzled binding | 1 | 0.062398725 |
| GO:0016248 | channel inhibitor activity | 1 | 0.062398725 |
| GO:0016831 | carboxy-lyase activity | 1 | 0.062398725 |
| GO:0017080 | sodium channel regulator activity | 1 | 0.062398725 |
| GO:0036002 | pre-mRNA binding | 1 | 0.062398725 |
| GO:1901681 | sulfur compound binding | 2 | 0.064225162 |
| GO:0001786 | phosphatidylserine binding | 1 | 0.067425053 |
| GO:0004601 | peroxidase activity | 1 | 0.074086276 |
| GO:0015485 | cholesterol binding | 1 | 0.074086276 |
| GO:0016903 | oxidoreductase activity, acting on the aldehyde or oxo group of donors | 1 | 0.075744373 |
| GO:0019825 | oxygen binding | 1 | 0.077399597 |
| GO:0016684 | oxidoreductase activity, acting on peroxide as acceptor | 1 | 0.079051952 |
