# Supplementary material for: Identification of Potential Biomarkers in Association With Progression and Prognosis in Epithelial Ovarian Cancer by Integrated Bioinformatics Analysis
Source: Front Genet. 2019 Oct 24;10:1031. doi: 10.3389/fgene.2019.01031 (PMC6822059; doi:10.3389/fgene.2019.01031)
Supplement: Supplementary file 7 [file Table_3.docx]

**Supplementary Table 3:** **KEGG pathway analysis of signiﬁcant molecule in cluster 1.**

| **ID** | **Term** | **Count** | **p-value** |
| --- | --- | --- | --- |
| hsa03030 | DNA replication | 1 | 0.023871578 |
| hsa04110 | Cell cycle | 1 | 0.080309303 |
| hsa04120 | Ubiquitin mediated proteolysis | 1 | 0.088420501 |
| hsa04218 | Cellular senescence | 1 | 0.102630829 |
| hsa05164 | Influenza A | 1 | 0.109364128 |
| hsa05206 | MicroRNAs in cancer | 1 | 0.18478652 |
