# Supplementary material for: Identification of Potential Biomarkers in Association With Progression and Prognosis in Epithelial Ovarian Cancer by Integrated Bioinformatics Analysis
Source: Front Genet. 2019 Oct 24;10:1031. doi: 10.3389/fgene.2019.01031 (PMC6822059; doi:10.3389/fgene.2019.01031)
Supplement: Supplementary file 8 [file Table_4.docx]

**Supplementary Table 4:** **GO analysis divided DEGs into three functional groups**

|  | **ID** | **Term** | **p-value** |
| --- | --- | --- | --- |
| BP | GO:0008283 | cell proliferation | 0.00694465 |
|  | GO:0001843 | neural tube closure | 0.01150272 |
|  | GO:0060672 | epithelial cell morphogenesis involved in placental branching | 0.018113567 |
|  | GO:0010046 | response to mycotoxin | 0.018113567 |
|  | GO:0001666 | response to hypoxia | 0.020796422 |
|  | GO:0045429 | positive regulation of nitric oxide biosynthetic process | 0.028069953 |
|  | GO:0000122 | negative regulation of transcription from RNA polymerase II promoter | 0.030971681 |
|  | GO:0031536 | positive regulation of exit from mitosis | 0.035902193 |
|  | GO:0098909 | regulation of cardiac muscle cell action potential | 0.047583607 |
|  | GO:0048672 | positive regulation of collateral sprouting | 0.047583607 |
|  | GO:0032092 | positive regulation of protein binding | 0.053042272 |
|  | GO:0042632 | cholesterol homeostasis | 0.057759561 |
|  | GO:0001775 | cell activation | 0.064843439 |
|  | GO:0006069 | ethanol oxidation | 0.070527604 |
|  | GO:0045944 | positive regulation of transcription from RNA polymerase II promoter | 0.074346954 |
|  | GO:0010952 | positive regulation of peptidase activity | 0.076177555 |
|  | GO:0003382 | epithelial cell morphogenesis | 0.081793497 |
|  | GO:0016477 | cell migration | 0.087208322 |
|  | GO:0006366 | transcription from RNA polymerase II promoter | 0.092262711 |
|  | GO:0070542 | response to fatty acid | 0.098439287 |
|  | GO:0030193 | regulation of blood coagulation | 0.098439287 |
| CC | GO:0005576 | extracellular region | 1.89E-04 |
|  | GO:0005615 | extracellular space | 0.002613968 |
|  | GO:0070062 | extracellular exosome | 0.007291278 |
|  | GO:0016323 | basolateral plasma membrane | 0.022972204 |
|  | GO:0016328 | lateral plasma membrane | 0.04008259 |
|  | GO:0009986 | cell surface | 0.044084961 |
|  | GO:0005829 | cytosol | 0.082108513 |
|  | GO:0044291 | cell-cell contact zone | 0.096996935 |
| MF | GO:0043565 | sequence-specific DNA binding | 2.68E-04 |
|  | GO:0030170 | pyridoxal phosphate binding | 0.045724868 |
|  | GO:0016504 | peptidase activator activity | 0.058260265 |
|  | GO:0046790 | virion binding | 0.058260265 |
|  | GO:0008483 | transaminase activity | 0.075073672 |
|  | GO:0042802 | identical protein binding | 0.079776335 |
|  | GO:0000981 | RNA polymerase II transcription factor activity, sequence-specific DNA binding | 0.083103938 |
