# Supplementary material for: Identification of Potential Biomarkers in Association With Progression and Prognosis in Epithelial Ovarian Cancer by Integrated Bioinformatics Analysis
Source: Front Genet. 2019 Oct 24;10:1031. doi: 10.3389/fgene.2019.01031 (PMC6822059; doi:10.3389/fgene.2019.01031)
Supplement: Supplementary file 9 [file Table_5.docx]

**Supplementary Table 5: survival analysis of 114 DEGs**

| **Gene** | **P value** |
| --- | --- |
| SLC4A11 | 0.001828 |
| CXCR4 | 0.004951 |
| IFI27 | 0.006858 |
| ADH1C | 0.006908 |
| KLHL14 | 0.007287 |
| TRIP13 | 0.008691 |
| TLCD1 | 0.016562 |
| ZWINT | 0.024433 |
| S100A2 | 0.02759 |
| PSAT1 | 0.030977 |
| ITLN1 | 0.032414 |
| LYPD1 | 0.038981 |
| FGF13 | 0.039604 |
| GPT2 | 0.051034 |
| NR2F1 | 0.055903 |
| ST6GALNAC1 | 0.057489 |
| EPCAM | 0.058935 |
| MECOM | 0.070878 |
| PCDH9 | 0.075535 |
| LPAR3 | 0.082168 |
| SPP1 | 0.085548 |
| SLC39A4 | 0.09867 |
| ISG15 | 0.1159 |
| DEFB1 | 0.136261 |
| TUBA4A | 0.137253 |
| POLR2H | 0.13949 |
| GADL1 | 0.156749 |
| FOLR1 | 0.168048 |
| HOXB2 | 0.178606 |
| SPINT2 | 0.1787 |
| FABP4 | 0.178975 |
| LRRC32 | 0.181276 |
| LYPD6B | 0.199004 |
| NANOG | 0.20887 |
| SCGB1D2 | 0.223699 |
| CTHRC1 | 0.225077 |
| EFEMP1 | 0.268082 |
| CD24 | 0.273694 |
| LGR6 | 0.276056 |
| CAV1 | 0.289832 |
| CNRIP1 | 0.30294 |
| GLDC | 0.304018 |
| CXXC5 | 0.3122 |
| EPHX4 | 0.315534 |
| ESM1 | 0.320666 |
| UCP2 | 0.321106 |
| PROCR | 0.324002 |
| EHF | 0.324862 |
| ARX | 0.336149 |
| TNNT1 | 0.339652 |
| KPNA2 | 0.344676 |
| CRABP2 | 0.378138 |
| MELK | 0.38269 |
| RNASE4 | 0.382859 |
| MEOX1 | 0.384703 |
| MYZAP | 0.40395 |
| ALDH1A1 | 0.415769 |
| WFDC2 | 0.439479 |
| MEIS2 | 0.454865 |
| FAM171A1 | 0.4604 |
| EYA2 | 0.481954 |
| HBB | 0.482802 |
| GPR160 | 0.490006 |
| CELF2 | 0.498404 |
| CCDC68 | 0.528185 |
| S100A4 | 0.530977 |
| HIST1H1C | 0.532035 |
| KIF11 | 0.542882 |
| PCOLCE2 | 0.544366 |
| SOX17 | 0.556568 |
| KIF15 | 0.589065 |
| CXCL16 | 0.595566 |
| ASS1 | 0.603188 |
| SYT4 | 0.61618 |
| ESRP1 | 0.621382 |
| KLK6 | 0.666061 |
| BACE2 | 0.676324 |
| RUNDC3B | 0.678831 |
| CSGALNACT1 | 0.681748 |
| CHCHD10 | 0.684126 |
| CDCA5 | 0.690943 |
| CP | 0.704547 |
| LHX2 | 0.711256 |
| SEL1L2 | 0.731928 |
| CLEC4M | 0.742352 |
| FZD10 | 0.743356 |
| TMEM150C | 0.749235 |
| INHBB | 0.7549 |
| PEG3 | 0.766208 |
| VGLL3 | 0.768813 |
| SCRIB | 0.782827 |
| FOXQ1 | 0.823653 |
| GRHL2 | 0.843801 |
| STC2 | 0.854744 |
| DLGAP5 | 0.857862 |
| MMP7 | 0.866925 |
| SCGB2A1 | 0.877044 |
| ABI3BP | 0.879697 |
| C21orf62 | 0.899321 |
| LCN2 | 0.900194 |
| BAMBI | 0.911726 |
| CLDN3 | 0.927716 |
| MPZL2 | 0.930045 |
| SFN | 0.93027 |
| RGS1 | 0.942617 |
| ASRGL1 | 0.943601 |
| FOXM1 | 0.948502 |
| MCM2 | 0.956051 |
| UBE2C | 0.963649 |
| PFKP | 0.978395 |
| PTX3 | 0.988189 |
| SNCAIP | 0.998792 |
| TMPRSS4 | 0.999564 |

DEGs, differentially expressed genes
